# Notes on vocalizations of Brazilian amphibians IV: advertisement calls of 20 Atlantic Forest frog species

**DOI:** 10.7717/peerj.7612

**Published:** 2019-09-13

**Authors:** Lucas Rodriguez Forti, Célio Fernando Baptista Haddad, Felipe Leite, Leandro de Oliveira Drummond, Clodoaldo de Assis, Lucas Batista Crivellari, Caio Marinho Mello, Paulo Christiano Anchietta Garcia, Camila Zornosa-Torres, Luís Felipe Toledo

**Affiliations:** 1 Instituto de Biologia, Universidade Federal da Bahia, Salvador, Bahia, Brazil; 2 Departamento de Zoologia and Centro de Aquicultura (CAUNESP), Universidade Estadual Paulista, Rio Claro, SP, Brazil; 3 Instituto de Ciências Biológicas e da Saúde, Universidade Federal de Viçosa, Florestal, MG, Brazil; 4 Centro de Biociências e Biotecnologia, Universidade Estadual Norte Fluminense, Campos dos Goytacazes, Rio de Janeiro, Brazil; 5 Programa de Pós-Graduação em Biologia Animal, Universidade Federal de Viçosa, Viçosa, Minas Gerais, Brazil; 6 Departamento de Zoologia, Universidade Federal do Paraná, Curitiba, Paraná, Brazil; 7 Programa de Pós-Graduação em Zoologia, Universidade Federal do Paraná, Curitiba, Paraná, Brazil; 8 Departamento de Zoologia, Universidade Federal de Minas Gerais, Belo Horizonte, Brazil; 9 Departamento de Biologia Animal, Universidade Estadual de Campinas, Campinas, SP, Brazil

**Keywords:** Bioacoustics, Animal behavior, Taxonomy, Atlantic forest, Amphibians, Anura, Conservation

## Abstract

Bioacoustics is a powerful tool used for anuran species diagnoses, given that advertisement calls are signals related to specific recognition and mate attraction. Thus, call descriptions can support species taxonomy. In spite of that, call descriptions are lacking for many species, delaying advances in biodiversity research. Here, we describe the advertisement calls of 20 anuran species from the Brazilian Atlantic Forest. We accessed 50 digital recordings deposited in the Fonoteca Neotropical Jacques Vielliard. Acoustic analyses were carried out in the software Raven pro 1.5. We provide a general comparison of call structure among species inside taxonomic groups and genera. The vocalizations described here belong to poorly known species, which are representatives of six families: Brachycephalidae, Bufonidae, Ceratophryidae, Cycloramphidae, Hylidae, and Phyllomedusidae. Despite this, still there are 163 species of anurans from Atlantic Forest with calls not formally described. Our work represents an important step in providing data for a taxonomic perspective and improving the knowledge of the Atlantic Forest anuran diversity.

## Introduction

Global biodiversity undergo a substantial crisis caused by human activities leading to a current rate of species extinctions tens to hundreds of times higher than the average across the past 10 million years (Ceballos et al., 2015; Tollefson, 2019). The research effort of several scientists all over the world have recognized several world hotspots, which are mainly based in endemism and loss of habitats (Myers et al., 2000). The Atlantic Forest is one of these biomes with special priority for conservation (Morellato & Haddad, 2000; Myers et al., 2000; Ribeiro et al., 2011). This Neotropical forest has been historically affected by deforestation, climate change, introduction of invasive species, and pandemic diseases (Bellard et al., 2014; Dean, 1996; Joly, Metzger & Tabarelli, 2014; Carvalho, Becker & Toledo, 2017; Forti et al., 2017a). It is a consensus that the first step for preserving biodiversity in nature is the formal recognition of species (Thomson et al., 2018). Such knowledge is the basis for promoting accurate inventories, field guides, lists of threatened species, and proper conservation management actions (Dijkstra, 2016). Unfortunately, the extraordinary biodiversity of the Atlantic Forest is not totally known and, considering the accelerated pace of deforestation (Ribeiro et al., 2009), an increase in the efforts of taxonomists for describing new species or reliable boundaries among taxa already described is urgent. The remarkable Atlantic Forest biota is a result of a complex diversification process generated by a rugged terrain, great habitat heterogeneity, and an ever-changing environment in the past (Carnaval et al., 2009; Toledo & Batista, 2012). This diversification process produced diverse levels of endemism (Toledo et al., 2014), which highlights even more the urgency for such taxonomical effort, since many species may be subjected to extinction before they are formally recognized.

Bioacoustics can be a powerful tool for solving taxonomic questions (Padial et al., 2010; Köhler et al., 2017). Many groups of animals produce sounds for attracting and selecting mates, so acoustic properties of such sexual signals are particularly rich and useful for species identification (Baker, 2001; Köhler et al., 2017). Although further evidence of its widespread use for taxonomy and as a phylogenetic signal is needed, we know, for instance, that in cases of syntopic species that use sounds to attract the reproductive partner, the recognition of boundaries among them is possible because vocalizations are frequently stereotyped (i.e., all individuals of a given species are able to emit similar vocalizations) and exposed to selective pressures, for example, to enhance mate recognition. Acoustic signals are essentially represented by three possible dimensions (a 3D gradient): power, time, and frequency (Snowdon, 2011). By applying a depth resolution (a detailed acoustic comparison), differences on these features usually permit a reliable distinction even between sister species. The vast majority of frog species are able to produce and hear sounds, which are their main way of communication (Duellman & Trueb, 1994; Narins, Feng & Fay, 2006; Wells, 2007). As territorial animals, male frogs vocalize from reproductive sites to advertise their reproductive status and space ownership (Wells & Schwartz, 2007), while females, which are auditively tuned to the band frequency of their own species (Fuzessery & Feng, 1982; Phelps, 2007), moves toward selected males. In such context, the advertisement call is an effective mechanism of prezygotic isolation (Blair, 1958; Schneider & Sinsch, 2007). This intraspecific signal has many stereotyped properties and, for such reason, advertisement calls of frogs are constantly used as a source of taxonomic and phylogenetic information (Littlejohn, 2001; Ryan & Rand, 2001; Robillard, Höbel & Gerhardt, 2006; Padial et al., 2010; Forti et al., 2017b).

An amplified knowledge of bioacoustics will improve the quality of long-term passive acoustic monitoring, which can be applied to understand spatial and temporal distribution of rare species in remote landscapes (Sugai et al., 2019). Once more species have calls formally described, knowledge gains new courses and novel approaches. The auditory detection, using automated recordings, supports both increase the knowledge of a poorly known species, as help us to monitor the impacts of human activities on biodiversity (Llusia et al., 2013; Schmeller et al., 2017). With the current fast advance of acoustic knowledge (Guerra et al., 2018), such techniques can also to be applied in favor of amphibian conservation.

Many scientists in the past made a substantial effort in recording Brazilian frogs, which allowed a considerable advance in the taxonomy of Neotropical anurans. We may cite as reference some remarkable works of Werner C.A. Bokermann and Adão J. Cardoso, which, summed, described the vocalizations of about 40 anuran species from the Atlantic Forest (Bokermann, 1964a, 1964b, 1966, 1967a, 1967b, 1967c, 1967d, 1972, 1973, 1974; Bokermann & Sazima, 1973; Sazima & Bokermann, 1978, 1982; Sazima & Cardoso, 1978; Cardoso & Haddad, 1984, 1990; Cardoso & Vielliard, 1985; García-Lopez, Heyer & Cardoso, 1996). Besides that, these two taxonomists recorded part of the acoustic material used in the present work, at a time when bioacoustics research required a great dedication, mainly by the transportation of heavy analog devices in the field. Our objective here was to improve the taxonomic and basic knowledge of Atlantic Forest anurans by describing, for the first time, vocalizations of 20 species of frogs. Besides, we are honoring W.C.A. Bokermann with the title of the manuscript, which symbolically represents the continuity of his three papers with similar titles (Bokermann, 1967a, 1967b, 1967c).

## Materials and Methods

We reviewed the literature, checking for species from the Atlantic Forest with calls already described, to produce a list of species with calls not formally described. After that, we obtained 50 audio files with advertisement calls of 20 species present in such list, by contacting researchers and surveying the files from the Fonoteca Neotropical Jacques Vielliard (FNJV, Unicamp, Campinas). These files came from different recordists, dates, localities, and equipments, which we specify in the Table 1. We identified species using specimens deposited in biological collections (Célio F.B. Haddad collection—CFBH, Museu de Zoologia João Moojen—MZUFV, Museu de História Natural Capão da Imbuia—MHNCI, Smithsonian Institution Washington—DC—US-Animalia, Museu de Zoologia da Universidade Estadual de Campinas “Adão José Cardoso”—ZUEC-AMP, Coleção de Anfíbios do Instituto Nacional da Mata Atlântica—MBML-Anfibios, Coleção de Anfíbios do Centro de Coleções Taxonômica da UFMG—UFMG-AMP, Coleção de Anfíbios do Museu de Ciências e Tecnologia da PUCRS—MCP), morphological evidence, and geographical distribution (see Table 1).

**Table 1:** Species, filtering configuration, sample data, and collection number of audio files analyzed.

Species	Bandpass filter (Hz)	Males	Calls analyzed	Localities	Recorder	Microphone	Recordist	Date	FNJV numbers	Identification criterion	Threat status by MMA (Ministério do Meio Ambiente) (2014)	Endemic to Atlantic Forest
Brachycephalidae												
*Ischnocnema concolor*	Below 400	7	63	Itamonte-MG	TASCAM DR-40		Leandro O. Drummond	29 November, 2016	36487–36496	Morphological evidence	Least concern	Yes
*Ischnocnema melanopygia*	Below 600	9	47	Itamonte-MG	TASCAM DR-40		Leandro O. Drummond	28 November, 2016 to 30 November, 2016	36497–36506, and 36508	Morphological evidence	Least concern	Yes
Bufonidae												
*Dendrophryniscus berthalutzae*		2	4	Treviso-SC	Marantz PMD-222	Audiotechnica AT 835b	Luis Felipe Toledo	25 January, 2006	12902 and 12903	Distribution and morphological evidence	Least concern	Yes
*Melanophryniscus alipioi*	Below 1,000	4	18	Ponta Grossa-PR	Marantz PMD 661	Yoga Ht 81	Caio Marinho Mello e Lucas Batista Crivellari	28 June, 2013	37450–37453	Distribution and morphological evidence and specimens deposited (MHNCI 1110411110)	Data deficient	Yes
*Melanophryniscus moreirae*	Below 1,200 and above 5,400	3	30	Itamonte-MG	Uher 4000 Report IC		Werner C.A. Bokermann	25 November, 1964 and 12 October, 1970	31783, 31785 and 32008	Distribution (type locality)	Least concern	Yes
*Melanophryniscus vilavelhensis*	Below 2,400	1	1	Ponta Grossa-PR	Marantz PMD-661	Yoga—Ht 81	Caio Marinho Mello e Lucas Batista Crivellari	5 August, 2016	33559	Distribution and morphological evidence and specimens deposited (MHNCI 10717 and 10718)	Least concern	Yes
Ceratoprhyidae												
*Ceratophrys aurita*	No filter	1	25	Linhares-ES	Uher 4000 Report IC		Werner C.A. Bokermann	9 November, 1964	31911	Specimens deposited (CFBH 26538; US-Animalia 241313; ZUEC-AMP 3623, 3801, 15838; MBML-Anfibios 2020, 3796, 3802)	Least concern	Yes
Cycloramphidae												
*Cycloramphus granulosus*	Below 200 above 3,000	1	3	São Jose do Barreiro-SP	Uher 4000 Report IC		Werner C.A. Bokermann	7 November, 1968	31950	Distribution and specimens deposited (US-Animalia 217903–217905)	Data deficient	Yes
*Cycloramphus izecksohni*	No filter	1	21	Corupá-SC	Nagra E		Célio F.B. Haddad	12 November, 1998	34035	Distribution and specimens deposited (CFBH 9394, 9395, 3773–3778, 10991, 10992, 10996, 10997)	Least concern	Yes
*Zachaenus parvulus*	No filter	1	5	Rio de Janeiro-RJ	Uher 4000 Report IC		Werner C.A. Bokermann	7 August, 1965	31926	Distribution (type locality)	Least concern	Yes
Hylidae												
*Boana guentheri*	Below 600 and above 5,500	1	3	Terra de Areia-RS	Nagra E	Sennheiser ME 66	Paulo Cristiano de Anchietta García	23 February, 1999	33063	Distribution and specimen deposited (ZUEC-AMP 11738)	Least concern	Yes
*Boana leptolineata*	No filter	4	169	São Francisco de Paula-RS and Lages-SC	Uher IC	M534	Adão José Cardoso	6 February, 1982, 11 February, 1982 and 15 October, 1995	30737–30739 and 31594	Distribution and specimen deposited (ZUEC-AMP 11252)	Least concern	Yes
*Bokermannohyla gouveai*	Above 5,000	1	31	Itamonte-MG	Marantz PMD222	Sennheiser ME 66	Célio F.B. Haddad	4 January, 2006	36485	Distribution (type locality)	Data deficient	Yes
*Ololygon flavoguttata*	Below 1,400 and above 6,000	1	29	Cataguases-MG	Tascam DR-40	Sennheiser ME66	Clodoaldo Lopes de Assis	31 July, 2016	36486	Specimens deposited (MZUFV 16022, 16495–16498, 17207–17210)	Least concern	Yes
*Ololygon tripui*		2	??	Alto-Caparaó-MG	Tascam DR-40	Built-in	Camila Zornosa Torres	27 July, 2016	32898 and 32389	Specimens deposited (ZUEC-AMP 23398, 23417, 24406, 23658, 24176, 23404, 24402, 22928, 23400, 23399, 22926, 23357, 24407, 24112, 23403, 23418)	Not Evaluated	Yes
Phyllomedusidae												
*Phasmahyla cochranae*	Below 675	2	35	São Jose do Barreiro-SP and Jundiaí-SP	Uher 4000 Report IC and Uher Monitor	M538	Werner C.A. Bokermann and Célio F.B. Haddad	3 November, 1965 and 4 October, 1988	31990 and 31152	Distribution (type locality)	Least concern	Yes
*Phasmahyla jandaia*	Below 850	2	7	Congonhas-MG and Santa Bárbara-MG	Marantz PMD 660	Sennheiser me-66	Felipe Leite	7 October, 2010 and 10 December, 2016	36507 and 36509	Specimens deposited (UFMG-GIR 360, 363, 375, 445, 296, 332, 1757, 1043, 1767, 376, 1044, 359, 301, 288, 377, 442, 354, 1765, 337, 1572, 14427, 17874)	Least concern	No
*Phrynomedusa appendiculata*	Below 855	1	2	Santo André-SP	Uher 4000 Report IC		Werner C.A. Bokermann	24 March, 1963	31849	Distribution and specimens deposited (ZUEC-AMP 15936; US-Animalia 162176)	Data deficient	Yes
*Phyllomedusa iheringii*	Below 450	1	18	Bagé-RS	Uher 4000 IC	M538	Adão José Cardoso	19 December, 1982	31156	Specimens deposited (MCP-Anfibios 1781; ZUEC-AMP 6244, 5311; UFMG-AMP 1630, 1631)	Least concern	No
*Pithecopus rusticus*	Below 1,000	3	20	Água Doce-SC	Marantz PMD 661	Yoga Ht 81	Caio Marinho Mello e Lucas Batista Crivellari	30 November, 2013	37454–37456	Distribution (type locality) and specimens deposited (MHNCI 10421 MHNCI 10422)	Not Evaluated	Yes

Before the acoustic analysis, we standardized all sound files to a pattern sample rate of 44.1 kHz and 16 bits of resolution saving the files in Audacity 2.1.1. For each species, we specified a band pass filter to decrease general background noise (Table 1). After the filtering process, calls were individually normalized (peak −0.8 dB) using Audacity 2.1.1 for avoiding biases related to the differences in intensity. We carried out acoustic analyses using the software Raven Pro 1.5 (Bioacoustics Research Program, 2011). For call selection, we used the waveform window. For spectral measurements, we adjusted a fast fourier transformation of 1,024 points, with a window of 50% overlap, temporal hope size of 256 samples, and grid spacing of 93.8 Hz. We used the note-centered approach (defining uninterrupted units of sound as notes and their entirety as a call) and the concepts of pulses, notes, and calls as defined by Köhler et al. (2017). Based in the terminology of Gerhardt & Huber (2002) and Wells (2007), we measured the following acoustic properties: (1) number of notes, (2) call/note duration, (3) number of pulses, (4) call rate, (5) harmonic structure, (6) rise time to the maximum amplitude, (7) range frequency, (8) minimum frequency, (9) maximum frequency, (10) fundamental frequency, and (11) dominant frequency. For such measurements we used the following functions: Bandwidth 90% (Hz) (for the range frequency, a band of frequency that includes 90% of the energy of the sound), Frequency 5% (Hz) (for the minimum frequency, ignoring 5% below the total energy in the selected call), Frequency 95% (Hz) (for the maximum frequency, ignoring 5% above the total energy in the selected call), Peak Frequency (Hz) (for dominant frequency), Delta Time (s) (for call/note duration), and Max Amplitude (U) (for finding the time to the maximum amplitude visualizing the limits in the waveform), available in the choose measurements menu in Raven (see also Köhler et al., 2017). We made descriptive statistics (mean, standard deviation, and range) based in individual measurements (by call and/or notes). When we had more than one male for each species we present mean and standard deviation based in mean values by males.

## Results

**Figure 1:**
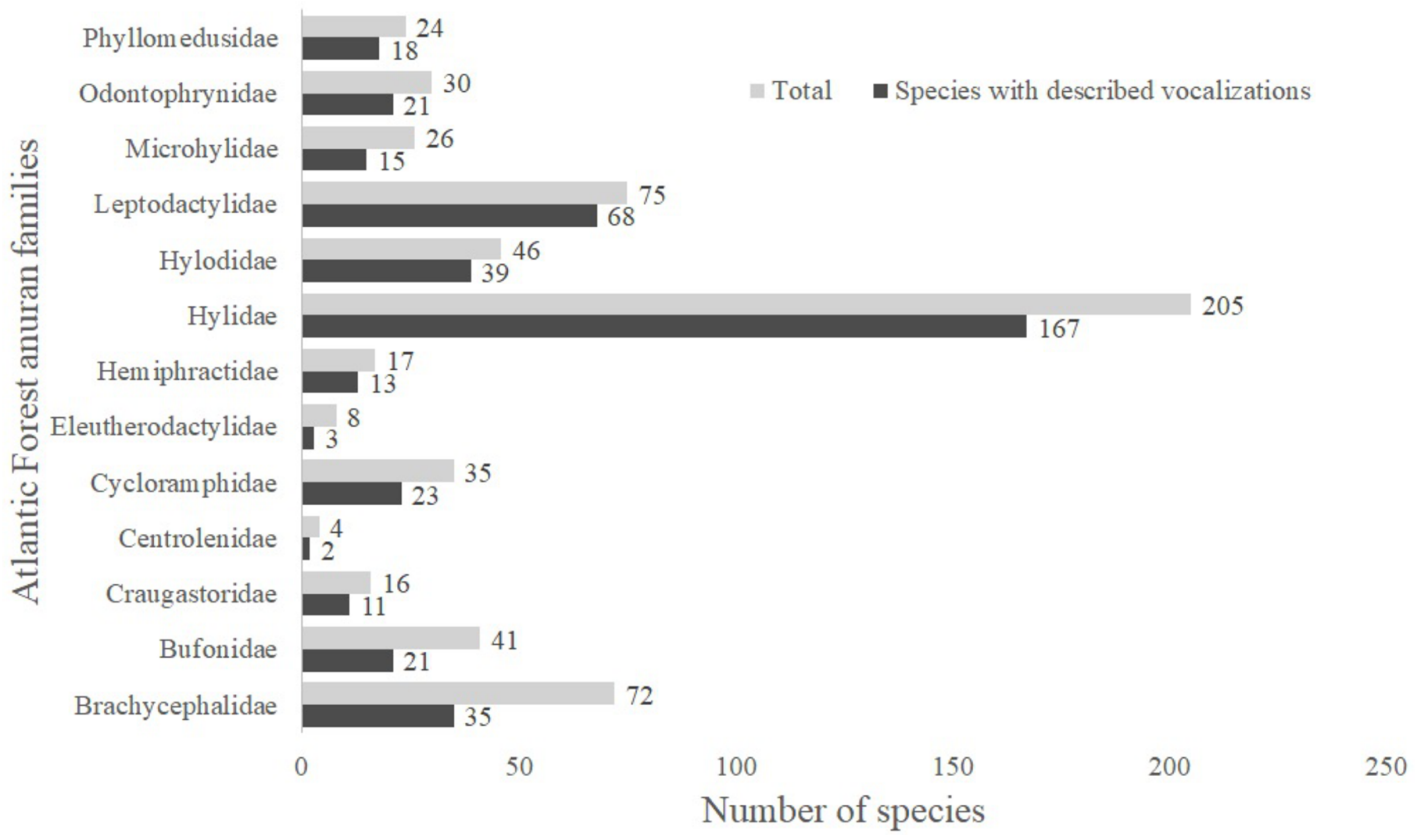
Number of species with described vocalizations from Brazilian Atlantic Forest anuran families.

A total of 13 Atlantic Forest frog families have species with unknown vocalizations (Fig. 1). Among these, we identified 163 species lacking vocalizations descriptions (nearly 26% of all 624 anuran species described up to April 2019 (L.F. Toledo, 2019, unpublished data); Table S1). Below we describe calls from 20 Atlantic Forest species of six different families.

BRACHYCEPHALIDAE
*Ischnocnema concolor* Targino, Costa, and Carvalho-e-Silva, 2009

**Figure 2:**
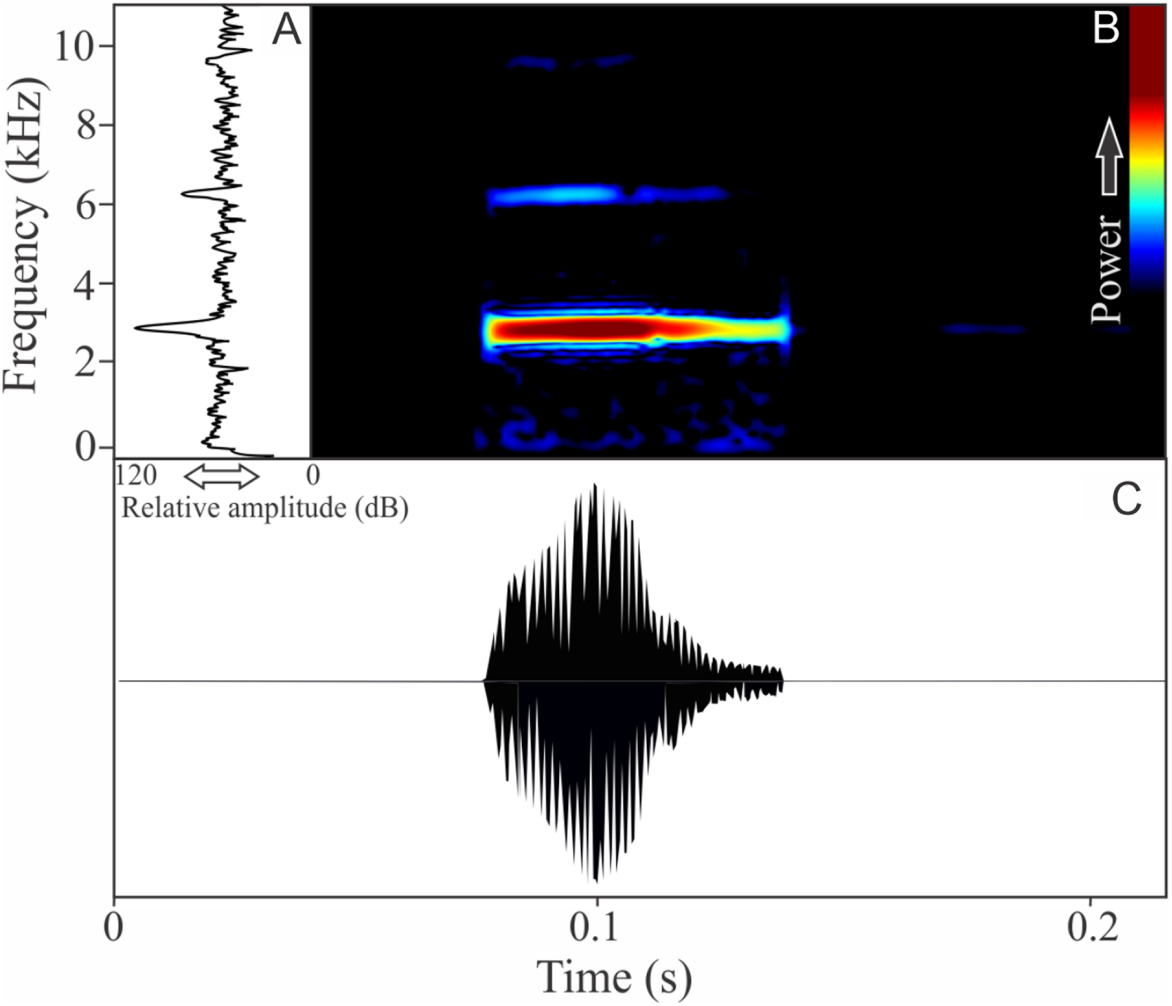
Amplitude spectra, spectrogram, and waveform of the advertisement call of *Ischnocnema concolor*. (A) Amplitude spectra (taken near to the midst of the call), (B) spectrogram, and (C) waveform of the advertisement call of *Ischnocnema concolor* from Parque Nacional do Itatiaia, Itamonte, state of Minas Gerais, Southeastern Brazil. Spectrogram window with DFT of 4096, grid spacing of 10.8 Hz and overlap of 75%.

The vocalization of *I. concolor* is composed by one non-pulsed harmonic note with 0.08 ± 0.01 s (ranging from 0.04 to 0.12 s, *n* = 63, males = 7) of duration. Sometimes, this note can be emitted as a series including two to four units with short regular intervals of 0.3 s. The call (note) occupy a strict mean range frequency of 103 ± 15 Hz (*n* = 63, males = 7), with a minimum frequency averaging 2,964 ± 98 Hz (ranging from 2,799 to 3,187 Hz, *n* = 63, males = 7) and maximum frequency of 3,064 ± 94 Hz (ranging from 2,929 to 3,273 Hz, *n* = 63, males = 7). The dominant frequency average is 3,019 ± 94 Hz (ranging from 2,842 to 3,230 Hz, *n* = 63, males = 7) and is located in the fundamental frequency band (Fig. 2). The second harmonic is up to 6 kHz. The rise time to the maximum amplitude is 0.022 ± 0.005 s (ranging from 0.013 to 0.038 s, *n* = 63, males = 7). Notes were emitted with an average interval of 5.7 ± 1.9 s (ranging from 0.24 to 18.4 s, *n* = 56, males = 7). Males called with a rate of 9.9 ± 2.6 calls/min (ranging from 6.7 to 14 calls/min, *n* = 7, males = 7).

*Ischnocnema melanopygia* Targino, Costa, and Carvalho-e-Silva, 2009

**Figure 3:**
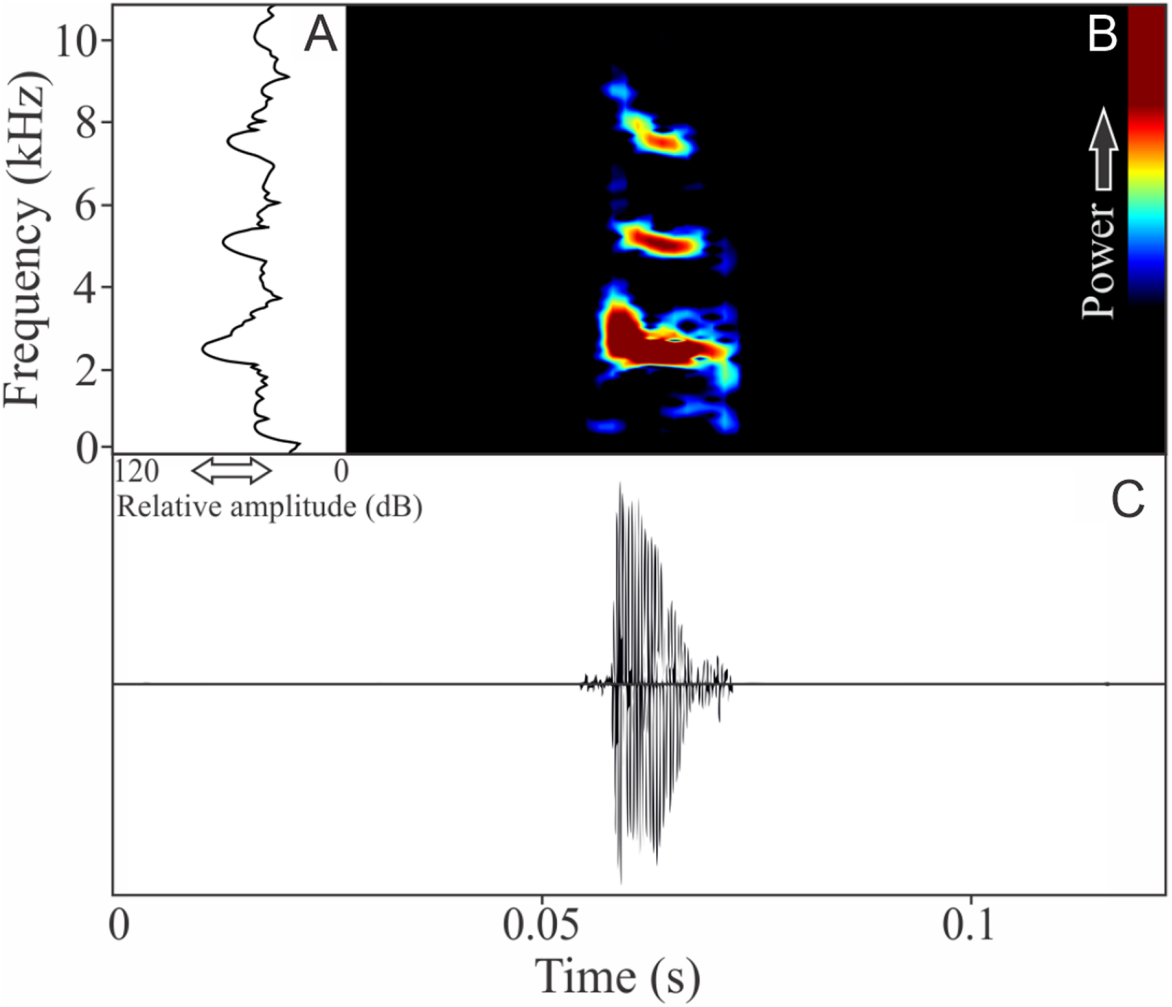
Amplitude spectra, spectrogram, and waveform of the advertisement call of *Ischnocnema melanopygia*. (A) Amplitude spectra (taken near to the midst of the call), (B) spectrogram, and (C) waveform of the advertisement call of *Ischnocnema melanopygia* from Parque Nacional do Itatiaia, Itamonte, state of Minas Gerais, Southeastern Brazil. Spectrogram window with DFT of 4096, grid spacing of 10.8 Hz and overlap of 75%.

This species has vocalizations composed by one to five non-pulsed harmonic notes with 0.024 ± 0.006 s (ranging from 0.01 to 0.04 s, *n* = 47, males = 9) of duration. Notes occupied a large mean range frequency of 1,109 ± 778 Hz (*n* = 47, males = 9), with a minimum frequency averaging 2,290 ± 122 Hz (ranging from 2,067 to 2,541 Hz, *n* = 47, males = 9) and maximum frequency of 3,399 ± 801 Hz (ranging from 2,326 to 5,211 Hz, *n* = 47, males = 9). The dominant frequency, located in the fundamental frequency band (Fig. 3), averages 2,407 ± 137 Hz (ranging from 2,153 to 2,756 Hz, *n* = 47, males = 9). The second and third harmonics varied between 4.8 and 5.4 kHz, and 7.2 and 8.0 kHz respectively. The rise time to the maximum amplitude is 0.004 ± 0.001 s (ranging from 0.002 to 0.009 s, *n* = 47, males = 9). Notes were emitted with an average interval of 3.81 ± 2.38 s (ranging from 0.16 to 30.81 s, *n* = 43, males = 9). Males called with a rate of 14.3 ± 4 notes/min (ranging from 7.5 to 20 notes/min, *n* = 9, males = 9).

BUFONIDAE
*Dendrophryniscus berthalutzae* (Bokermann, 1962)

**Figure 4:**
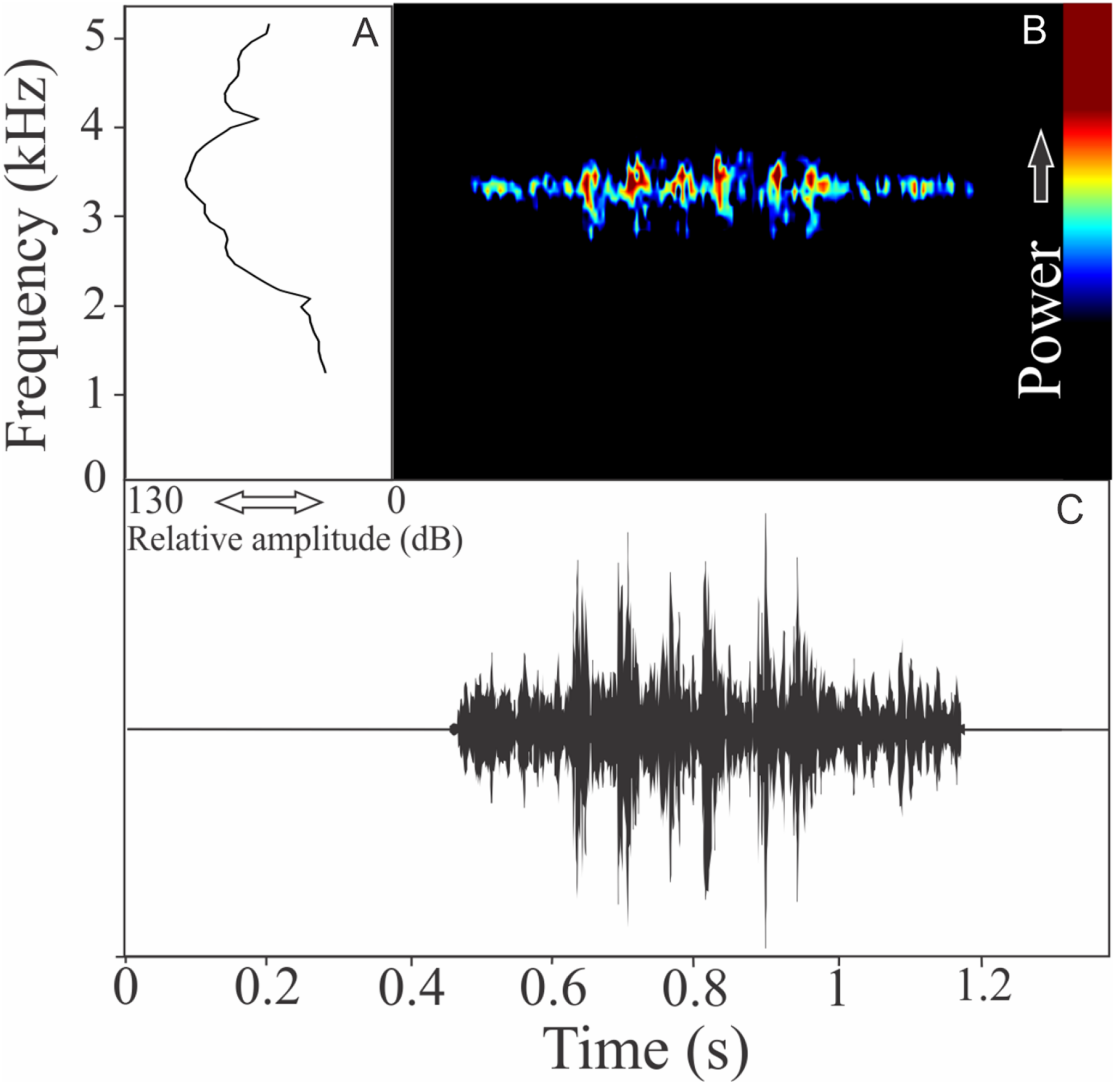
Amplitude spectra, spectrogram, and waveform of the advertisement call of *Dendrophryniscus brevipollicatus*. (A) Amplitude spectra (taken near to the midst of the call), (B) spectrogram, and (C) waveform of the advertisement call of *Dendrophryniscus brevipollicatus* from Treviso, state of Santa Catarina, Southern Brazil. Spectrogram window with DFT of 4096, grid spacing of 10.8 Hz and overlap of 75%.

The vocalization of *D. berthalutzae* is a sequence of six pulsed notes (Fig. 4), with duration of 0.43 ± 0.069 s (ranging from 0.37 to 0.57 s, *n* = 4, males = 2), and rise time to the maximum amplitude of 0.187 ± 0.006 s (ranging from 0.08 to 0.29 s, *n* = 4, males = 2). There is no harmonic structure and each note is composed by five to eight pulses with 0.029 ± 0.009 s (ranging from 0.017 to 0.042 s, *n* = 4, males = 2) of duration. The more intense note has a range frequency averaging 700 ± 15 Hz (ranging from 646 to 745 Hz, *n* = 4, males = 2), with mean of minimum frequency of 2,907 ± 0.04 Hz (ranging from 2,842 to 2,972 Hz, *n* = 4, males = 2), and maximum frequency of 3,607 ± 15 Hz (ranging from 3,575 to 3,618, *n* = 4, males = 2). The dominant frequency of the more intense note is 3,391 ± 15 Hz (ranging from 3,316 to 3,488 Hz, *n* = 4, males = 2).

*Melanophryniscus alipioi* Langone, Segalla, Bornschein, and de Sá, 2008

**Figure 5:**
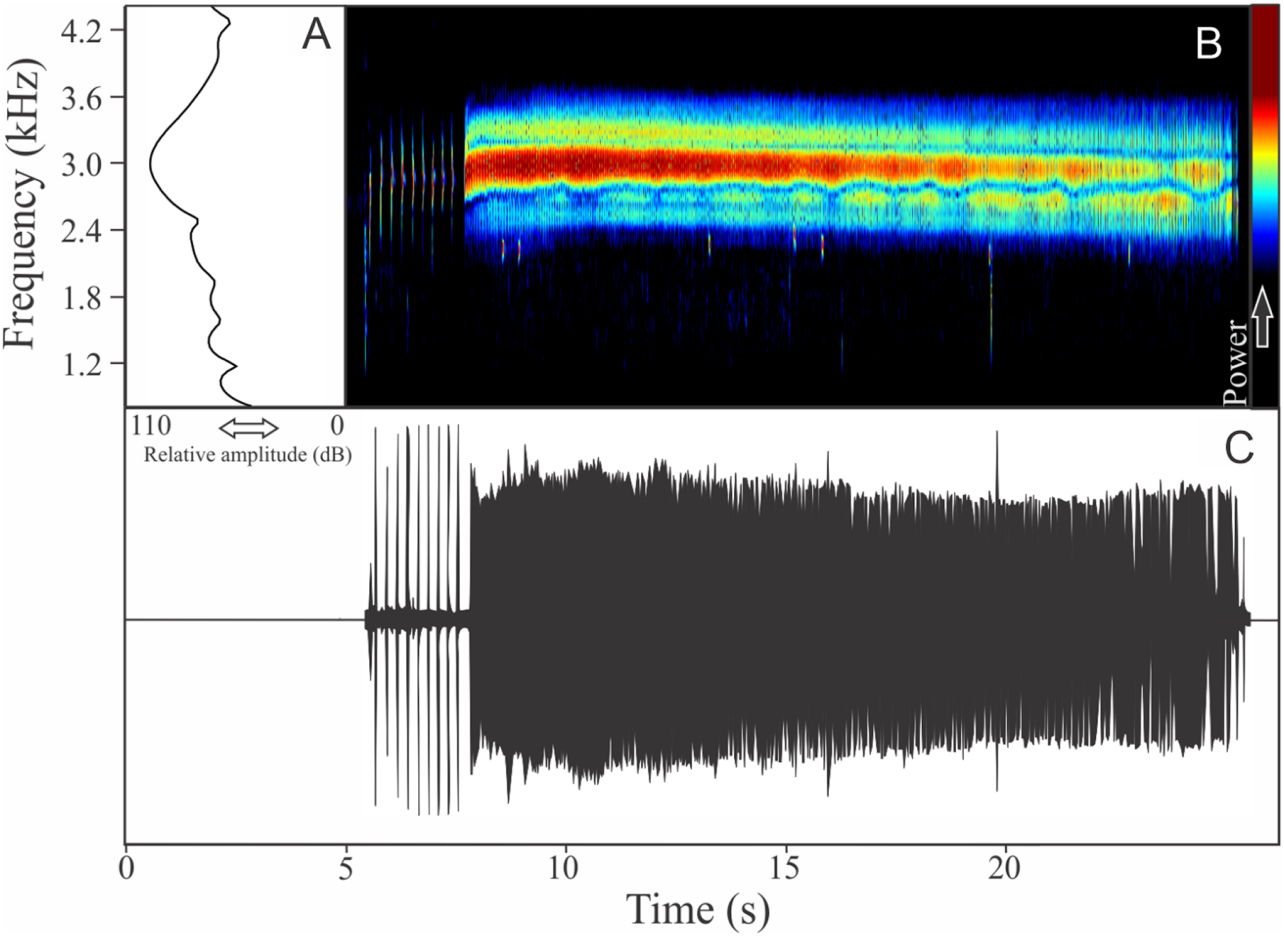
Amplitude spectra, spectrogram, and waveform of the advertisement call of *Melanophryniscus alipioi*. (A) Amplitude spectra (taken near to the midst of the call), (B) spectrogram, and (C) waveform of the advertisement call of *Melanophryniscus alipioi* from Campina Grande do Sul, state of Paraná, Southern Brazil. Spectrogram window with DFT of 4096, grid spacing of 10.8 Hz and overlap of 75%.

This species has a vocalization composed of two segments consisting of series of notes with harmonics (825 ± 165, *n* = 18, males = 4). The first segment, which we determined as an introductory segment, has a note series composed by longer notes with larger silence intervals than the main segment (Fig. 5). Including both segments, the call duration is 17.7 ± 3.9 s (ranging from 6.7 to 25.8 s, *n* = 18, males = 4). Each isolated note from the introductory section has mean duration of 0.046 ± 0.012 s (ranging from 0.025 to 0.060 s, *n* = 18, males = 4) and silence interval between notes of 0.172 ± 0.036 s (ranging from 0.124 to 0.234, *n* = 18, males = 4). Notes in the main segment have a mean duration of 0.007 ± 0.001 s (ranging from 0.006 to 0.010 s, *n* = 18, males = 4) and silence interval between notes of 0.009 ± 0.001 s (ranging from 0.006 to 0.013 s, *n* = 18, males = 4). Notes in the introductory segment show a mean range frequency of 352 ± 127 Hz (*n* = 18, males = 4), with minimum frequency averaging 2,411 ± 200 Hz (ranging from 2,196 to 2,799 Hz, *n* = 18, males = 4), maximum frequency of 2,763 ± 260 Hz (ranging from 2,433 to 3,058 Hz, *n* = 18, males = 4), and dominant frequency of 2,664 ± 248 Hz (ranging from 2,240 to 2,929 Hz, *n* = 18, males = 4). The rise time to the maximum amplitude in the first segment is 0.0185 ± 0.00811 s (ranging from 0.005 to 0.036 s, *n* = 18, males = 4). Notes in the main segment have a mean range frequency of 561 ± 262 Hz (*n* = 18, males = 4), with minimum frequency averaging 2,490 ± 257 Hz (ranging from 2,182 to 2,842 Hz, *n* = 18, males = 4), maximum frequency of 3,051 ± 321 Hz (ranging from 2,598 to 3,531 Hz, *n* = 18, males = 4), and dominant frequency of 2,759 ± 229 Hz (ranging from 2,440 to 2,972 Hz, *n* = 18, males = 4). The rise time to the maximum amplitude in the second segment is 0.0026 ± 0.0007 s (ranging from 0.001 to 0.004 s, *n* = 18, males = 4). Both types of notes have harmonic structure with a fundamental frequency of the same value of dominant frequency.

*Melanophryniscus moreirae* (Miranda-Ribeiro, 1920)

**Figure 6:**
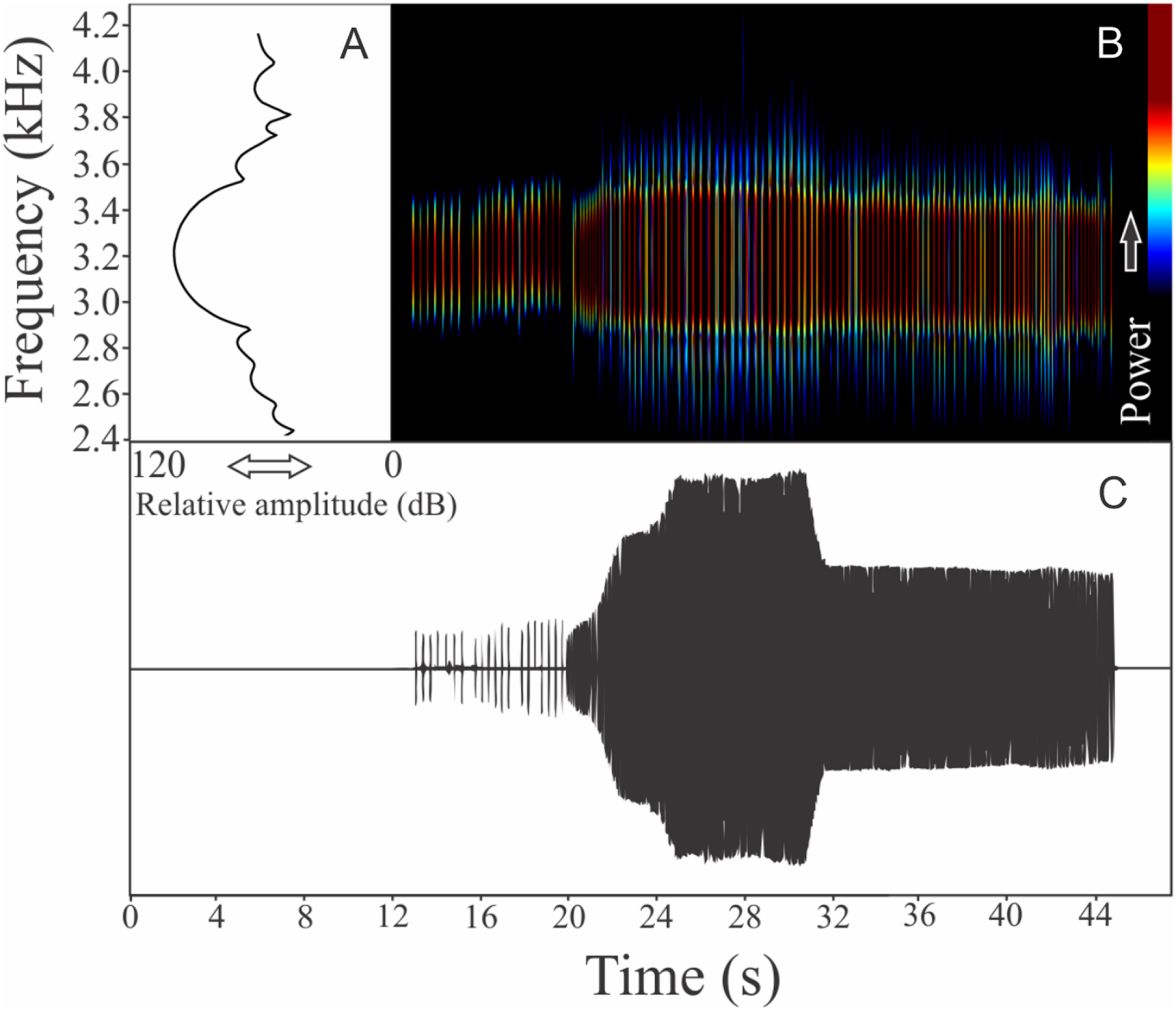
Amplitude spectra, spectrogram, and waveform of the advertisement call of *Melanophryniscus moreirae*. (A) Amplitude spectra (taken near to the midst of the cal—complex call black line and simple call blue line), (B) spectrogram, and (C) waveform of the advertisement call of *Melanophryniscus moreirae* from Parque Nacional do Itatiaia, Itamonte, state of Minas Gerais, Southeastern Brazil. Spectrogram window with DFT of 4096, grid spacing of 10.8 Hz and overlap of 75%.

We recognized two different sections of vocalizations of *M. moreirae*: a non-harmonic note series (non-pulsed notes repeated in regular interval), which is more commonly emitted and another part composed by an isolated and non-pulsed harmonic note (Fig. 6). It is possible that the note series is the advertisement call, while isolated harmonic notes are aggressive calls. The note series is a repetition of 70 ± 47 notes (ranging from six to 300 notes, *n* = 14, males = 2), which have 1.83 ± 1.26 s (ranging from 0.21 to 6.08, *n* = 14, males = 2) of duration. Each note of the note series call has 0.014 ± 0.004 s (ranging from 0.010 to 0.022 s, *n* = 10, males = 2) of duration, while the call composed by an isolated note averages 0.086 ± 0.032 s (ranging from 0.038 to 0.140 s, *n* = 16, males = 2) of duration. The more intense note in the note series call vary among the 3rd and 57th unit, with rise time to the maximum amplitude achieved in 0.62 ± 0.70 s (ranging from 0.07 to 3.07 s, *n* = 14, males = 2). Range frequency of the note series call is 252 ± 9 Hz with minimum frequency averaging 1,733 ± 15 Hz (ranging from 1,637 to 1,809 Hz, *n* = 14, males = 2), maximum frequency of 1,985 ± 6 Hz (ranging from 1,938 to 2,153 Hz, *n* = 14, males = 2), and dominant frequency of 1,856 ± 67 Hz (ranging from 1,766 to 1,938 Hz, *n* = 14, males = 2). The aggressive call (isolated notes) show range frequency of 169 ± 4 Hz, with minimum frequency averaging 1,717 ± 70 Hz (ranging from 1,594 to 1,809 Hz, *n* = 16, males = 2), maximum frequency of 1,886 ± 74 Hz (ranging from 1,680 to 2,196 Hz, *n* = 16, males = 2), and dominant frequency (=fundamental frequency) of 1,809 ± 91 Hz (ranging from 1,637 to 1,895 Hz, *n* = 16, males = 2). The second harmonic is about 3.5 kHz in the aggressive call. The rise time to the maximum amplitude of this aggressive note is 0.025 ± 0.010 s (ranging from 0.012 to 0.049 s, *n* = 16, males = 2).

*Melanophryniscus vilavelhensis* Steinbach-Padilha, 2008

**Figure 7:**
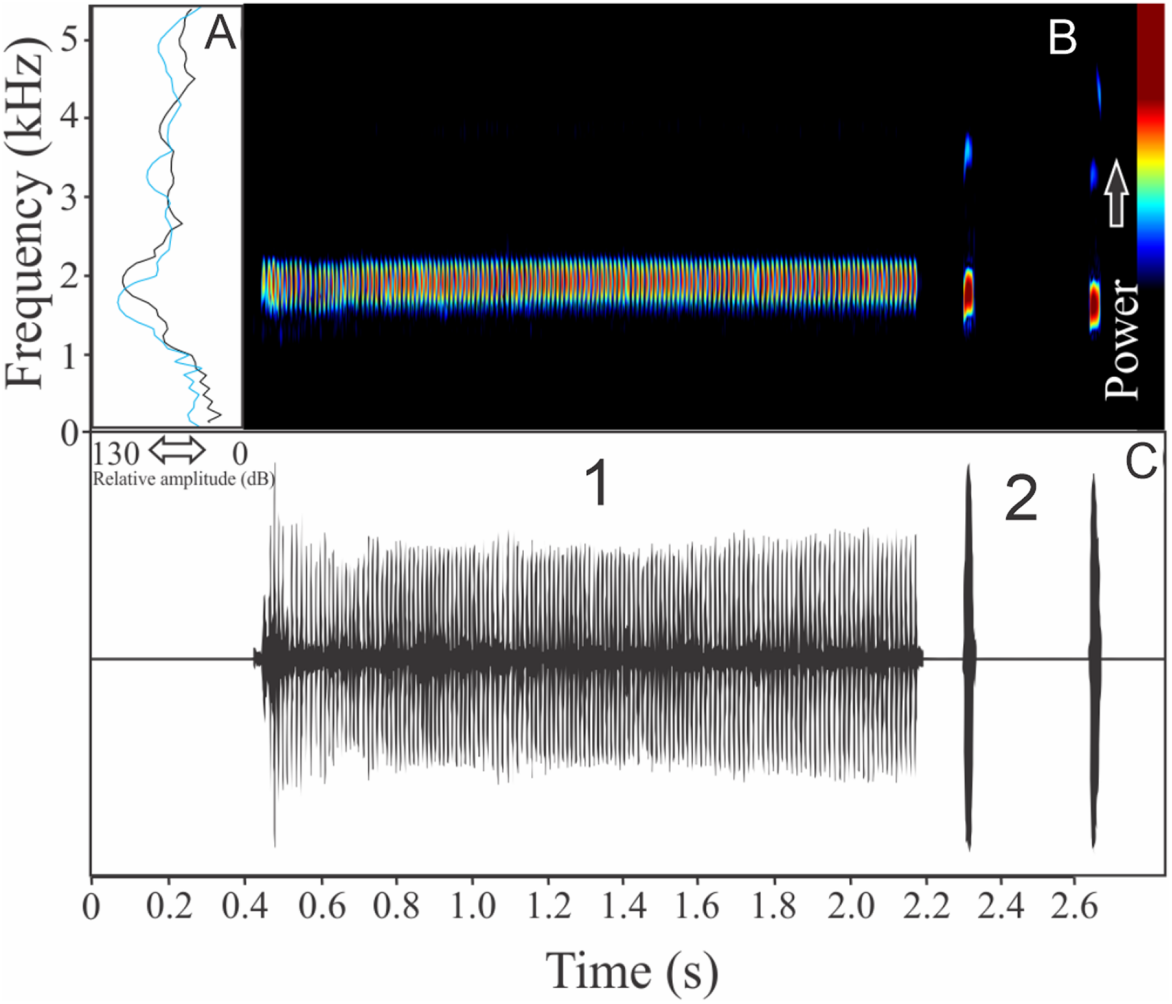
Amplitude spectra, spectrogram, and waveform of the advertisement call of *Melanophryniscus vilavelhensis*. (A) Amplitude spectra (taken near to the midst of the call), (B) spectrogram, and (C) waveform of the advertisement call of *Melanophryniscus vilavelhensis* from Ponta Grossa, state of Paraná, Southern Brazil. Spectrogram window with DFT of 4096, grid spacing of 10.8 Hz and overlap of 75%.

This species has the vocalization composed of two segments of non-harmonic note series (546 notes, *n* = 1). Notes are not pulsed. As in *M. alipioi*, the first segment (determined as introductory segment) has a note series composed by longer notes with larger silence intervals than the main segment (Fig. 7). Each isolated note from the introductory section has a mean duration of 0.07 ± 0.008 s (ranging from 0.06 to 0.08 s, *n* = 8) and silence interval between notes of 0.24 ± 0.01 s (ranging from 0.23 to 0.26, *n* = 7). The rise time to the maximum amplitude in this section is 6.4 s. Notes in the main segment have a mean duration of 0.013 ± 0.001 s (ranging from 0.02 to 0.03 s, *n* = 8) and silence interval between notes of 0.03 ± 0.002 s (ranging from 0.02 to 0.03 s, *n* = 7). Notes in the introductory segment show a range frequency of 86.1 Hz, with minimum frequency averaging 3,182 ± 36 Hz (ranging from 3,144 to 3,230 Hz, *n* = 8), maximum frequency of 3,268 ± 36 Hz (ranging from 3,230 to 3,316 Hz, *n* = 8), and dominant frequency of 3,230 ± 40 Hz (ranging from 3,187 to 3,273 Hz, *n* = 8). The first segment notes in the main segment have a range frequency of 188 Hz, with minimum frequency averaging 3,128 ± 22 Hz (ranging from 3,101 to 3,144 Hz, *n* = 8), maximum frequency of 3,316 ± 0 Hz (*n* = 8), and dominant frequency of 3,230 ± 0 Hz (*n* = 8). The rise time to the maximum amplitude in the main section is 10.6 s. Notes do not have harmonic structure and the rise time to the maximum amplitude considering segments combined is 17.5 s.

CERATOPHRYIDAE
*Ceratophrys aurita* (Raddi, 1823)

**Figure 8:**
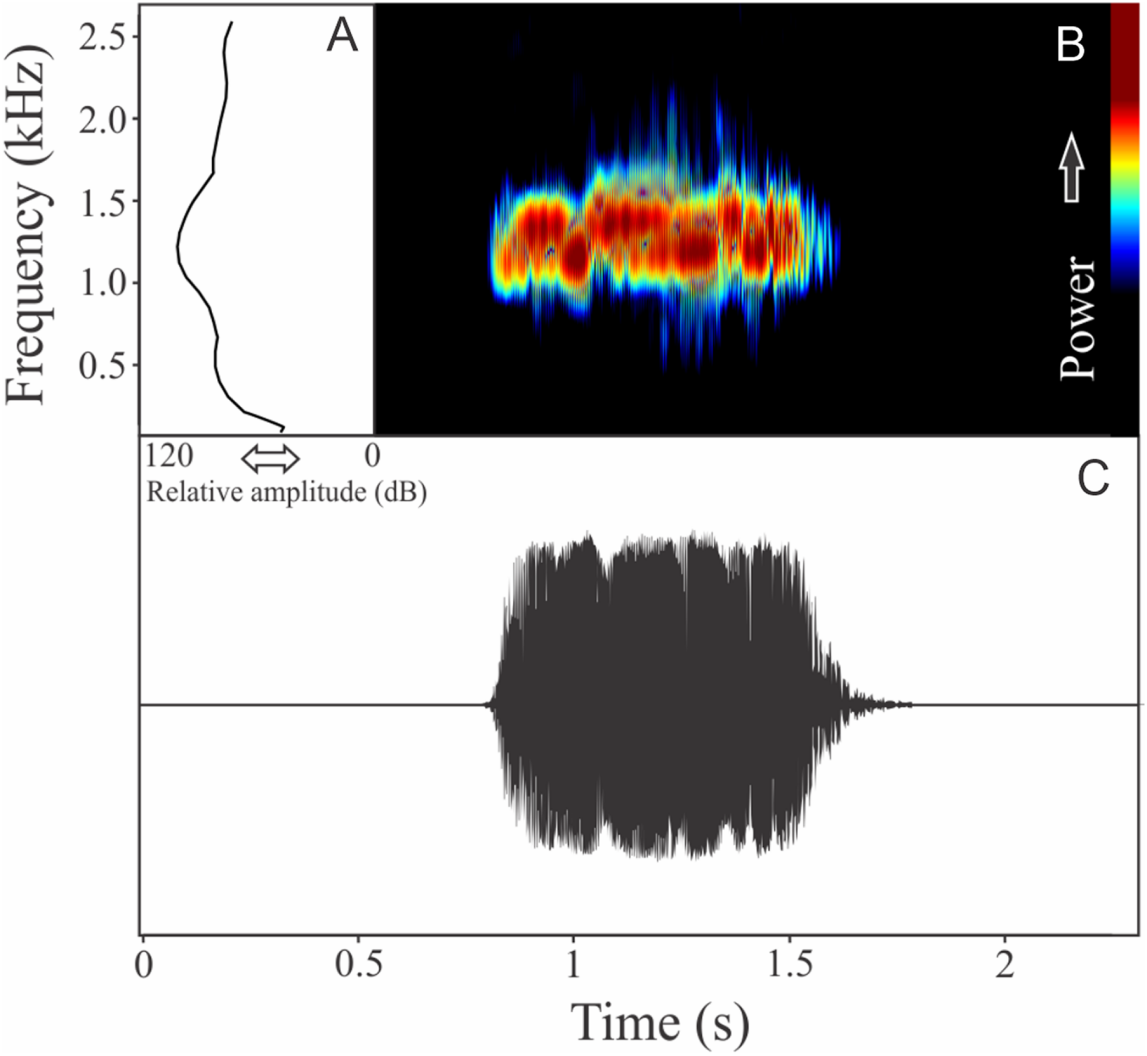
Amplitude spectra, spectrogram, and waveform of the advertisement call of *Ceratophrys aurita*. (A) Amplitude spectra (taken near to the midst of the call), (B) spectrogram, and (C) waveform of the advertisement call of *Ceratophrys aurita* from Linhares, state of Espírito Santo, Southeastern Brazil. Spectrogram window with DFT of 4096, grid spacing of 10.8 Hz and overlap of 75%.

The vocalization of *Ceratophrys aurita* is a single note with a mean of 149 ± 19 fused pulses (ranging from 80 to 172, *n* = 25). Each call has an average of 0.87 ± 0.09 s (ranging from 0.54 to 0.98 s, *n* = 25), repeated in an interval of 1.23 ± 1.11 s (ranging from 0.11 to 4.95 s, *n* = 25), and call rate of 27 calls/min. Notes have an unstable spectral modulation, with upward and downward pattern along the call (Fig. 8). Despite some weak sidebands being visible using lower contrasts in spectrogram window, the range frequency is not so large, occupying 479 ± 129 Hz, with minimum frequency averaging 1,044 ± 107 Hz (ranging from 603 to 1,163 Hz, *n* = 25), maximum frequency of 1,523 ± 46 Hz (ranging from 1,421 to 1,550 Hz, *n* = 25), and peak dominant frequency of 1,261 ± 69 Hz (ranging from 1,163 to 1,421 Hz, *n* = 25). The rise time to the maximum amplitude is 0.39 ± 0.18 s (ranging from 0.06 to 0.70 s, *n* = 25).

CYCLORAMPHIDAE
*Cycloramphus granulosus* Lutz, 1929

**Figure 9:**
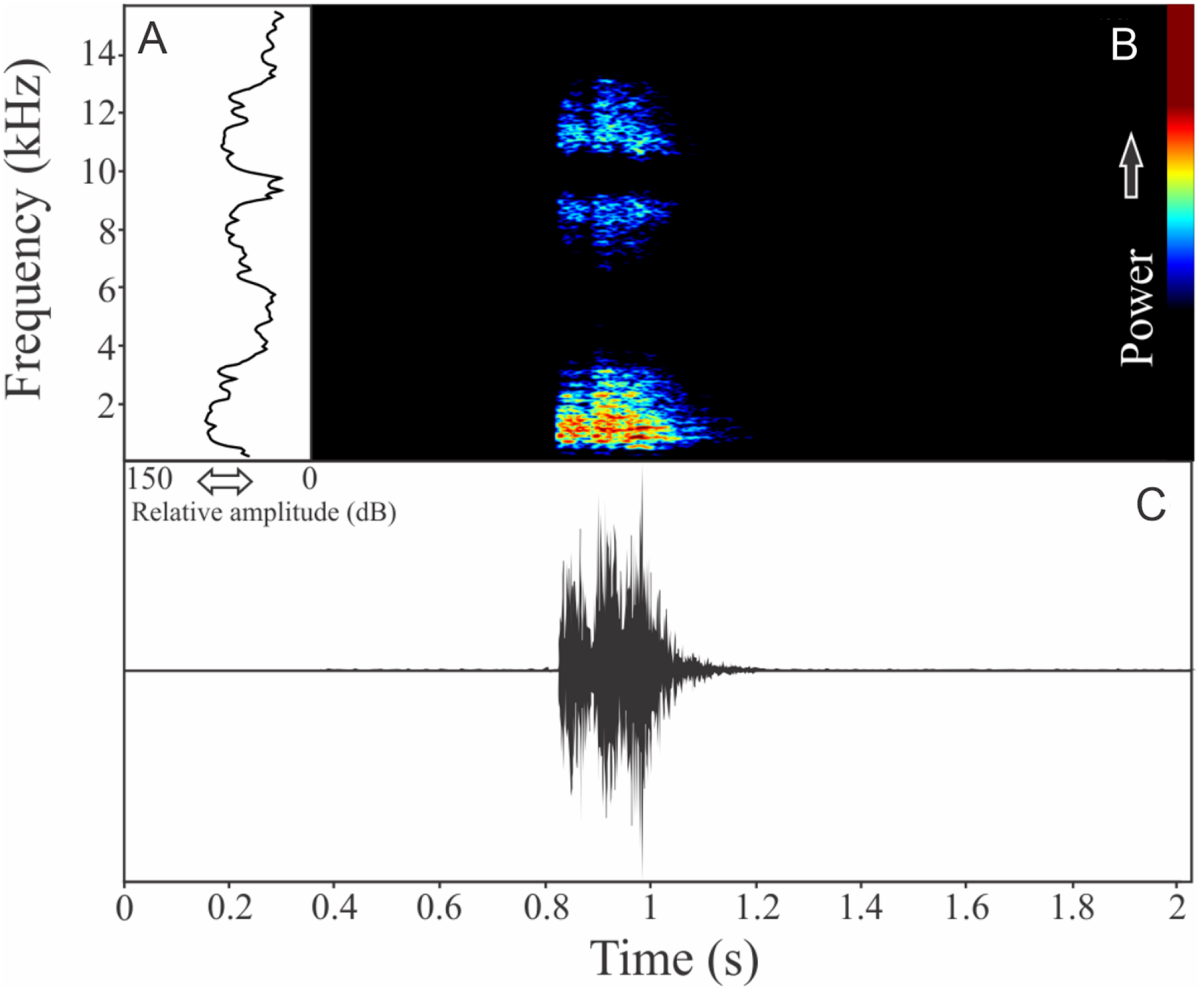
Amplitude spectra, spectrogram, and waveform of the advertisement call of *Cycloramphus granulosus*. (A) Amplitude spectra (taken near to the midst of the call), (B) spectrogram, and (C) waveform of the advertisement call of *Cycloramphus granulosus* from São José do Barreiro, state of São Paulo, Southeastern Brazil. Spectrogram window with DFT of 4096, grid spacing of 10.8 Hz and overlap of 75%.

This species has a vocalization composed of a single pulsed and harmonic note. The note duration is 1.16 ± 0.25 s (ranging from 0.97 to 1.44 s, *n* = 3), and the number of pulses varied among 25 and 33 pulses. The harmonic structure appears more clearly related to the more intense pulses (Fig. 9). This call has a large range frequency of 1,321 ± 66 Hz, which is particularly affected by the energy distribution between the first (fundamental) and second harmonics. Minimum frequency averaged 804 ± 25 Hz (ranging from 775 to 818 Hz, *n* = 3), maximum frequency was 2,125 ± 66 Hz (ranging from 2,067 to 2,196 Hz, *n* = 3), and dominant frequency (= fundamental frequency) was 1,263 ± 50 Hz (ranging from 1,206 to 1,292 Hz, *n* = 3). The second harmonic is about 2 kHz. The rise time to the maximum amplitude is 0.91 ± 0.30 s (ranging from 0.70 to 1.25 s, *n* = 3).

*Cycloramphus izecksohni* Heyer, 1983

**Figure 10:**
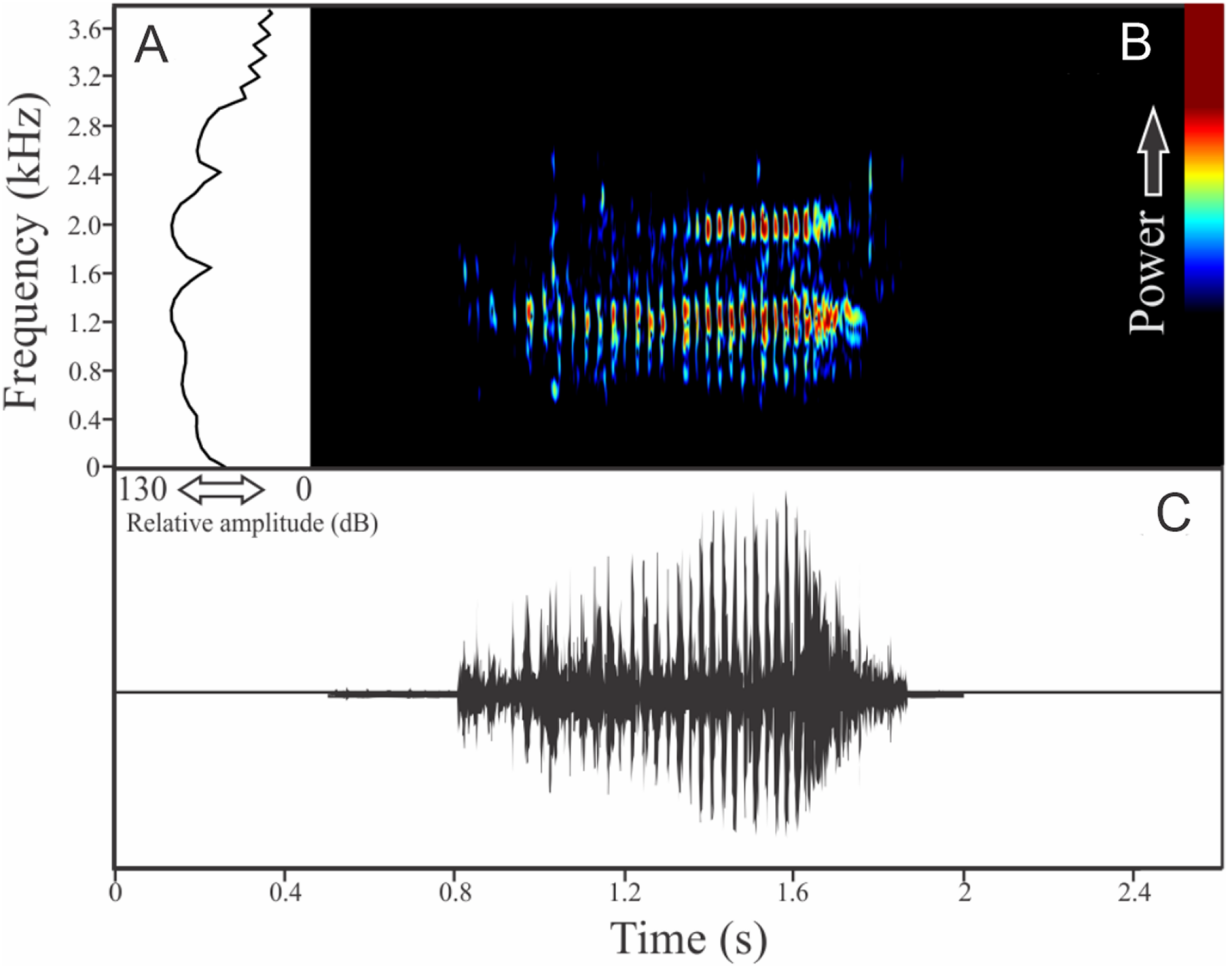
Amplitude spectra, spectrogram, and waveform of the advertisement call of *Cycloramphus izecksohni*. (A) Amplitude spectra (taken near to the midst of the call), (B) spectrogram, and (C) waveform of the advertisement call of *Cycloramphus izecksohni* from Corupá, state of Santa Catarina, Southern Brazil. Spectrogram window with DFT of 4096, grid spacing of 10.8 Hz and overlap of 75%.

The recording of *Cycloramphus izecksohni* was obtained based in a male recorded inside a plastic bag. The vocalization is a single note with one to three pulses. Sometimes two notes are quickly repeated (interval of 0.1 s). The call duration is 0.28 ± 0.05 s (ranging from 0.17 to 0.36 s, *n* = 21). The call rate was 20 calls/min, repeated with an interval of 2.25 ± 1.01 s (ranging from 0.09 to 4.80 s, *n* = 21). A harmonic structure is present with a large spectral interval between the first and the second harmonics (Fig. 10). The dominant frequency is the first harmonic (= fundamental frequency), which has a peak of 1,140 ± 265 Hz (ranging from 861 to 1,594 Hz, *n* = 21). The range frequency may be excessively high considering the harmonic distribution, which includes 6,936 ± 2,573 Hz. Minimum frequency averages 681 ± 216 Hz (ranging from 43 to 818 Hz, *n* = 21) and maximum frequency is 7,617 ± 2,649 Hz (ranging from 2,153 to 10,422 Hz, *n* = 21). The second harmonic is up to eight kHz. The rise time to the maximum amplitude is 0.09 ± 0.05 s (ranging from 0.03 to 0.21 s, *n* = 21).

*Zachaenus parvulus* (Girard, 1853)

**Figure 11:**
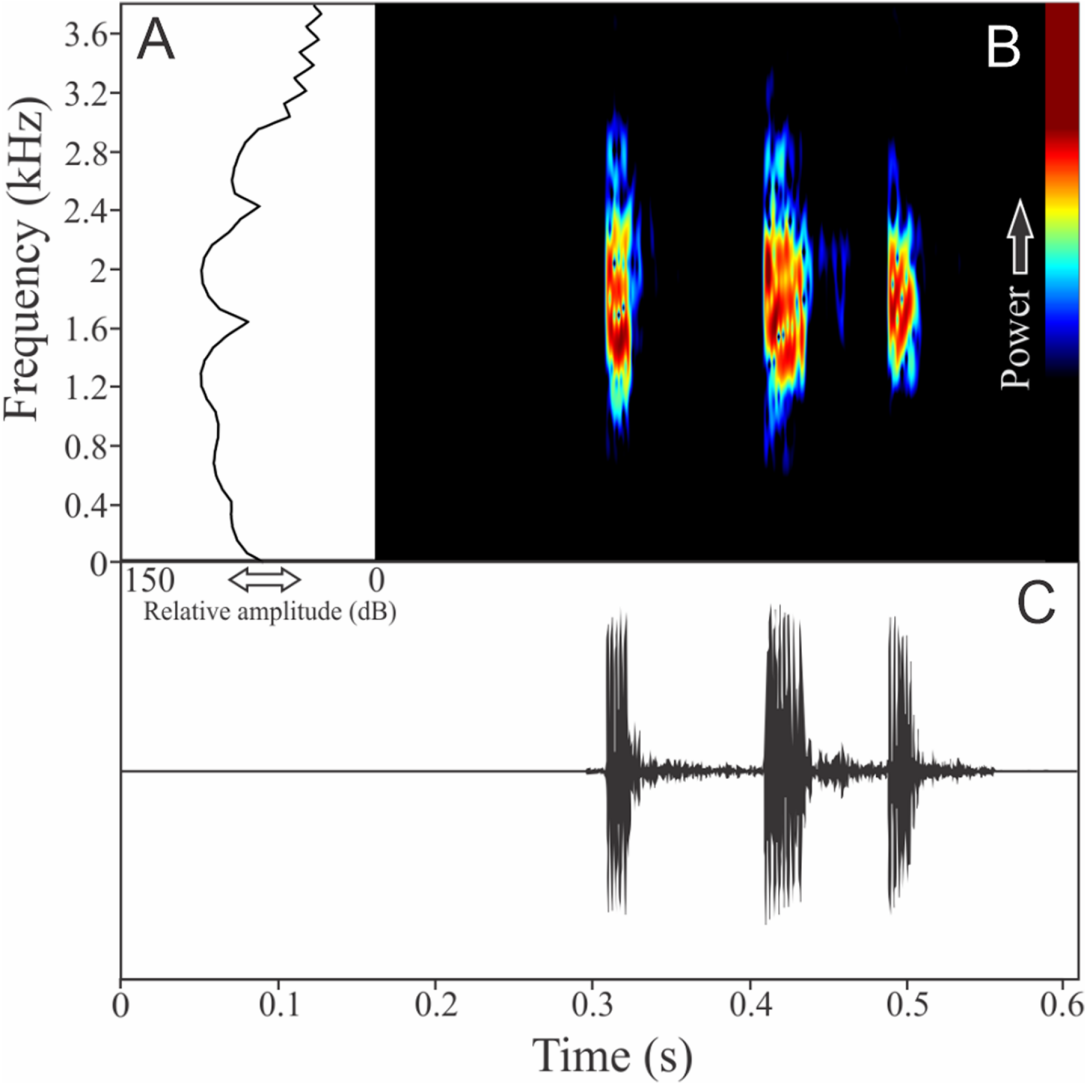
Amplitude spectra, spectrogram, and waveform of the advertisement call of *Zachaenus parvulus*. (A) Amplitude spectra (taken near to the midst of the call), (B) spectrogram, and (C) waveform of the advertisement call of *Zachaenus parvulus* from Rio de Janeiro, state of Rio de Janeiro, Southeastern Brazil. Spectrogram window with DFT of 4096, grid spacing of 10.8 Hz and overlap of 75%.

Two to three notes compose the vocalization of *Zachaenus parvulus* (Fig. 11). These notes are similar, having 7 ± 4 pulses (ranging from three to 14, *n* = 13) with duration of 0.03 ± 0.01 s (ranging from 0.02 to 0.04 s, *n* = 5). The call duration is 0.19 ± 0.04 s (ranging from 0.14 to 0.23 s, *n* = 5) and the more intense note is generally the second (80%). The range frequency is 2,188 ± 56 Hz, with minimum frequency averaging 138 ± 56 Hz (ranging from 86 to 215 Hz, *n* = 5), maximum frequency of 2,326 ± 53 Hz (ranging from 2,240 to 2,369 Hz, *n* = 5), and dominant frequency of 1,525 ± 163 Hz (ranging from 1,378 to 1,766 Hz, *n* = 5). The rise time to the maximum amplitude is 0.08 ± 0.05 s (ranging from 0.0 to 0.11 s, *n* = 5).

HYLIDAE
*Boana guentheri* (Boulenger, 1886)

**Figure 12:**
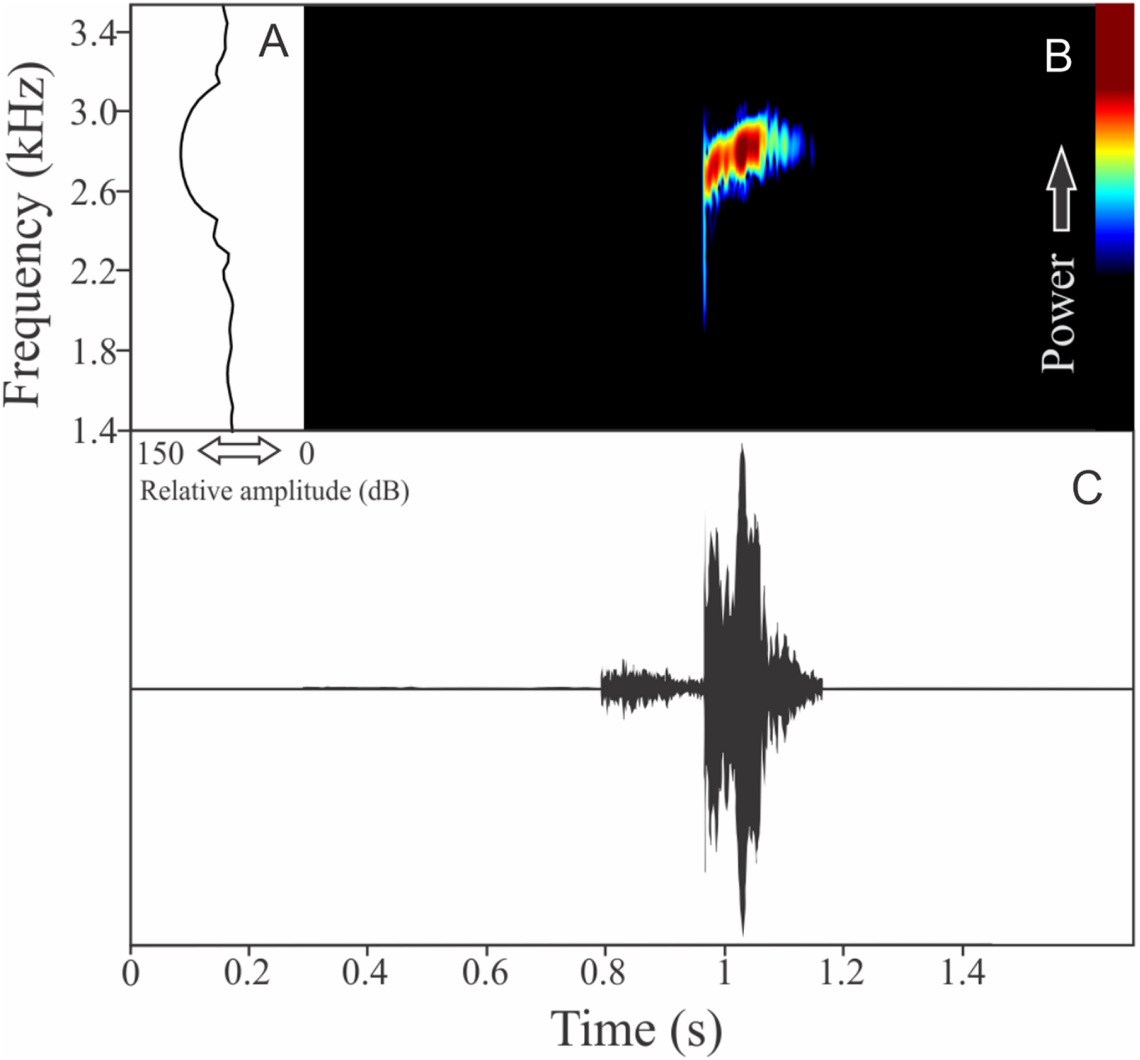
Amplitude spectra, spectrogram, and waveform of the advertisement call of *Boana guentheri*. (A) Amplitude spectra (taken near to the midst of the call), (B) spectrogram, and (C) waveform of the advertisement call of *Boana guentheri* from Terra de Areia, state of Rio Grande do Sul, Southern Brazil. Spectrogram window with DFT of 4096, grid spacing of 10.8 Hz and overlap of 75%.

The vocalization of *Boana guentheri* is an upward single frequency modulated note (Fig. 12). The note has one to two pulses with duration of 0.17 ± 0.02 s (ranging from 0.15 to 0.19 s, *n* = 3). The range frequency is 172 ± 43 Hz, with minimum frequency averaging 2,570 ± 108 Hz (ranging from 2,455 to 2,670 Hz, *n* = 3), maximum frequency of 2,742 ± 90 Hz (ranging from 2,670 to 2,842 Hz, *n* = 3), and dominant frequency of 2,699 ± 90 Hz (ranging from 2,627 to 2,799 Hz, *n* = 3). The rise time to the maximum amplitude is 0.09 ± 0.03 s (ranging from 0.07 to 0.12 s, *n* = 3).

*Boana leptolineata* (Braun & Braun, 1977)

**Figure 13:**
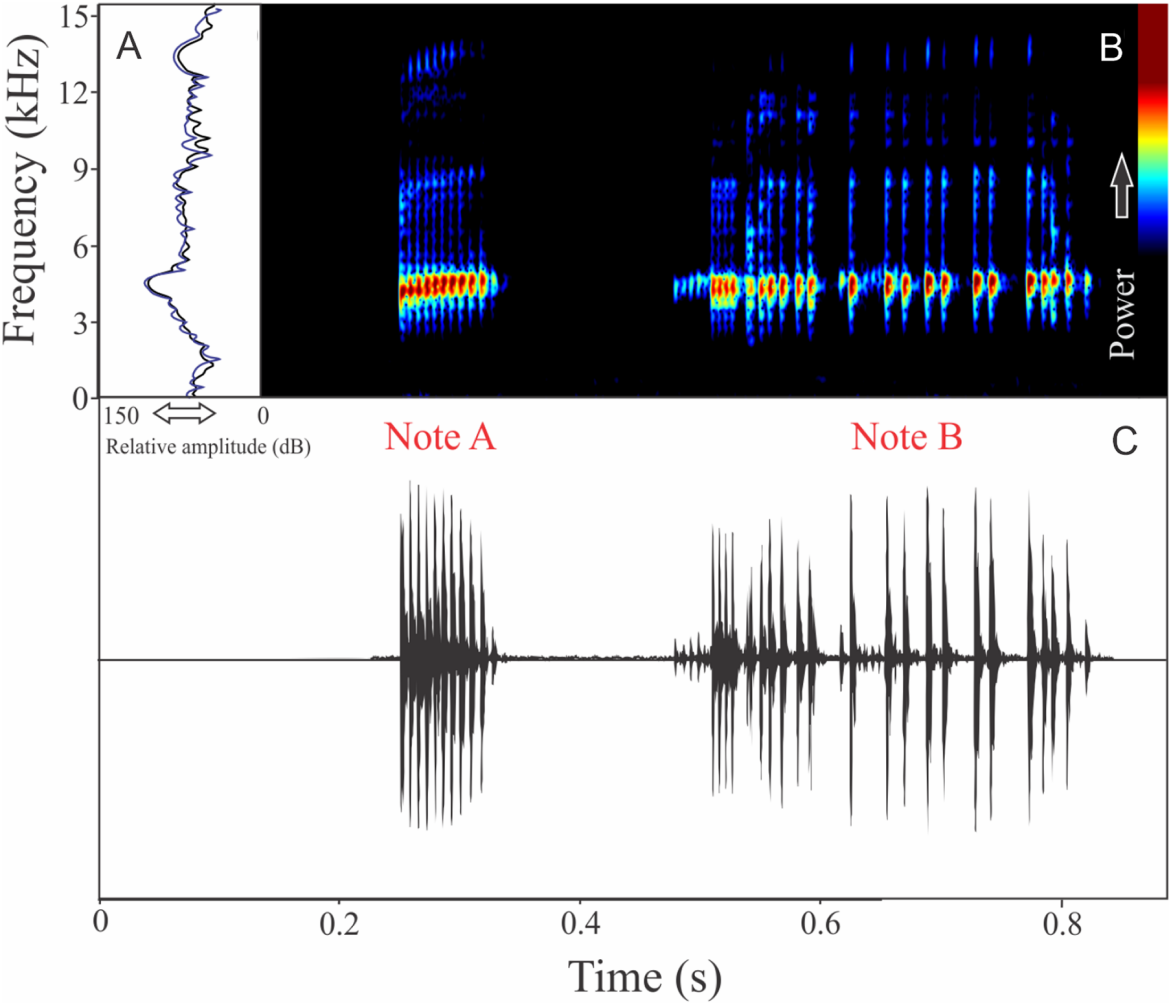
Amplitude spectra, spectrogram, and waveform of the advertisement call of *Boana leptolineata*. (A) Amplitude spectra (taken near to the midst of the call), (B) spectrogram, and (C) waveform of the advertisement call of *Boana leptolineata* from Lages, state of Santa Catarina, Southern Brazil. Spectrogram window with DFT of 4096, grid spacing of 10.8 Hz and overlap of 75%.

We identified two different notes in the vocalization of *Boana leptolineata*. Note “A” is a train of fused pulses (varying from two to 14), sounding as a trill, while the note “B” is a sequence of pulses (varying from four to 50) with discrete silence interval, sounding as many click-like units (Fig. 13). Both notes have harmonic structure. These notes are frequently combined in a sequence, composing complex calls. It is possible that these notes have different social functions. Note “A” duration is 0.06 ± 0.02 s (ranging from 0.02 to 0.16 s, *n* = 122, males = 4), with rise time to the maximum amplitude of 0.016 ± 0.005 s (ranging from 0 to 0.10 s, *n* = 122, males = 4). The range frequency is 499 ± 98 Hz, with minimum frequency averaging 4,005 ± 352 Hz (ranging from 3,402 to 4,651 Hz, *n* = 122, males = 4), maximum frequency of 4,504 ± 394 Hz (ranging from 3,919 to 5,082 Hz, *n* = 122, males = 4), and dominant frequency (= fundamental frequency) of 4,244 ± 368 Hz (ranging from 3,575 to 4,823 Hz, *n* = 122, males = 4). Note “B” duration is 0.391 ± 0.063 s (ranging from 0.19 to 0.64 s, *n* = 47, males = 4), with rise time to the maximum amplitude of 0.250 ± 0.062 s (ranging from 0.01 to 0.42 s, *n* = 47, males = 4). The range frequency is 665 ± 294 Hz, with minimum frequency averaging 3,915 ± 361 Hz (ranging from 2,972 to 4,436 Hz, *n* = 47, males = 4), maximum frequency of 4,580 ± 484 Hz (ranging from 3,919 to 6,546 Hz, *n* = 47, males = 4), and dominant frequency (= fundamental frequency) of 4,268 ± 415 Hz (ranging from 3,790 to 4,867 Hz, *n* = 47, males = 4). Second harmonic, for both notes, is up to 8,000 Hz.

*Bokermannohyla gouveai* (Peixoto & Cruz, 1992)

**Figure 14:**
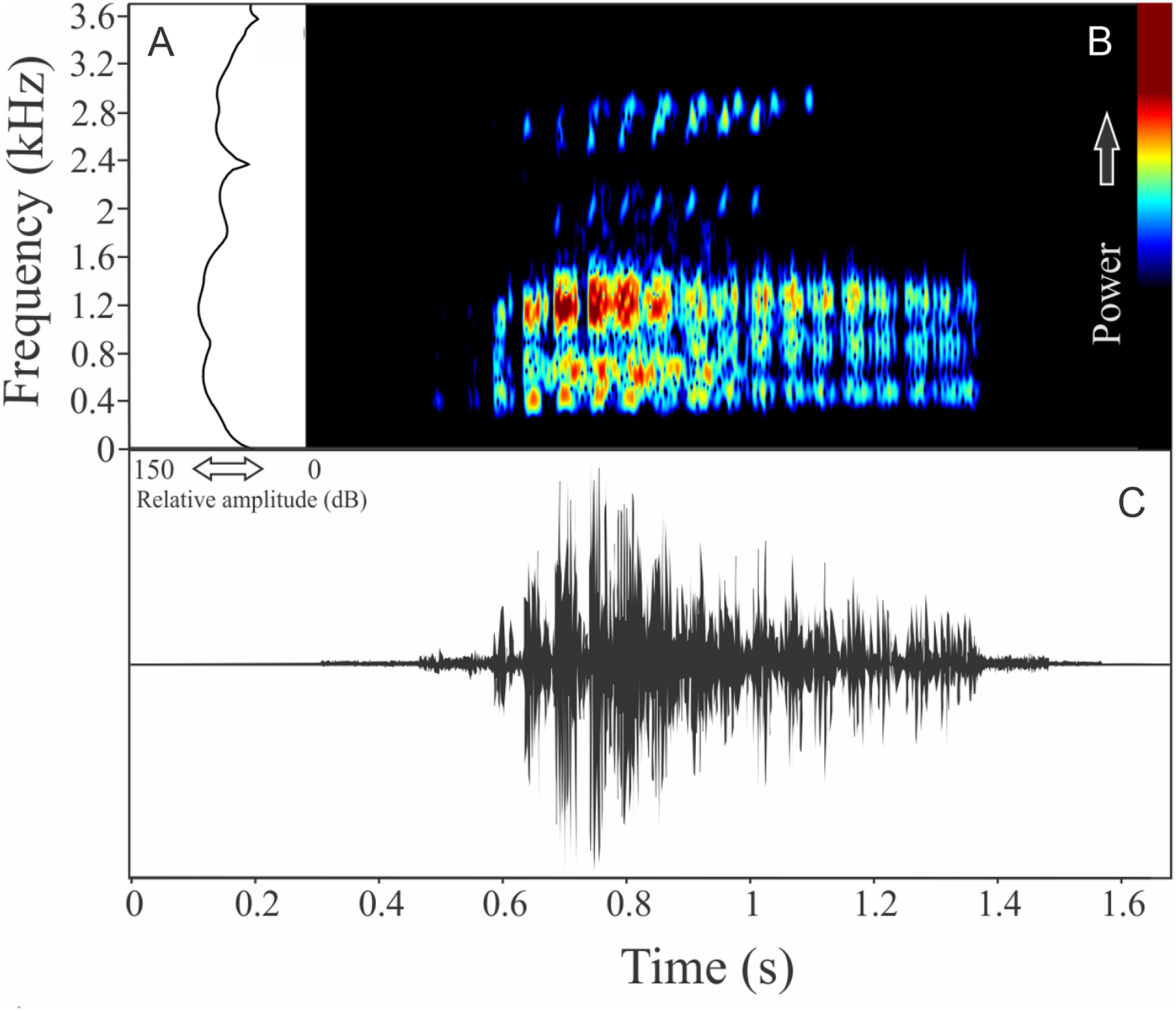
Amplitude spectra, spectrogram, and waveform of the advertisement call of *Bokermannohyla gouveai*. (A) Amplitude spectra (taken near to the midst of the call), (B) spectrogram, and (C) waveform of the advertisement call of *Bokermannohyla gouveai* from Parque Nacional do Itatiaia, Itamonte, state of Minas Gerais, Southeastern Brazil. Spectrogram window with DFT of 4096, grid spacing of 10.8 Hz and overlap of 75%.

The vocalization of *Bokermannohyla gouveai* is a single harmonic note composed by five to 15 pulses (Fig. 14). Call duration is 0.42 ± 0.12 s (ranging from 0.27 to 0.80 s, *n* = 31), with rise time to the maximum amplitude of 0.18 ± 0.07 s (ranging from 0 to 0.34 s, *n* = 31). The recorded male emitted a sequence of calls at a rate of 24 calls/min. The range frequency is 860 ± 119 Hz, with minimum frequency averaging 596 ± 140 Hz (ranging from 431 to 1,077 Hz, *n* = 31), maximum frequency of 1,456 ± 75 Hz (ranging from 1,335 to 1,594 Hz, *n* = 31), and dominant frequency (= fundamental frequency) of 1,127 ± 201 Hz (ranging from 560 to 1,378 Hz, *n* = 31). The second harmonic is about 2,800 Hz.

*Ololygon flavoguttata* (Lutz & Lutz, 1939)

**Figure 15:**
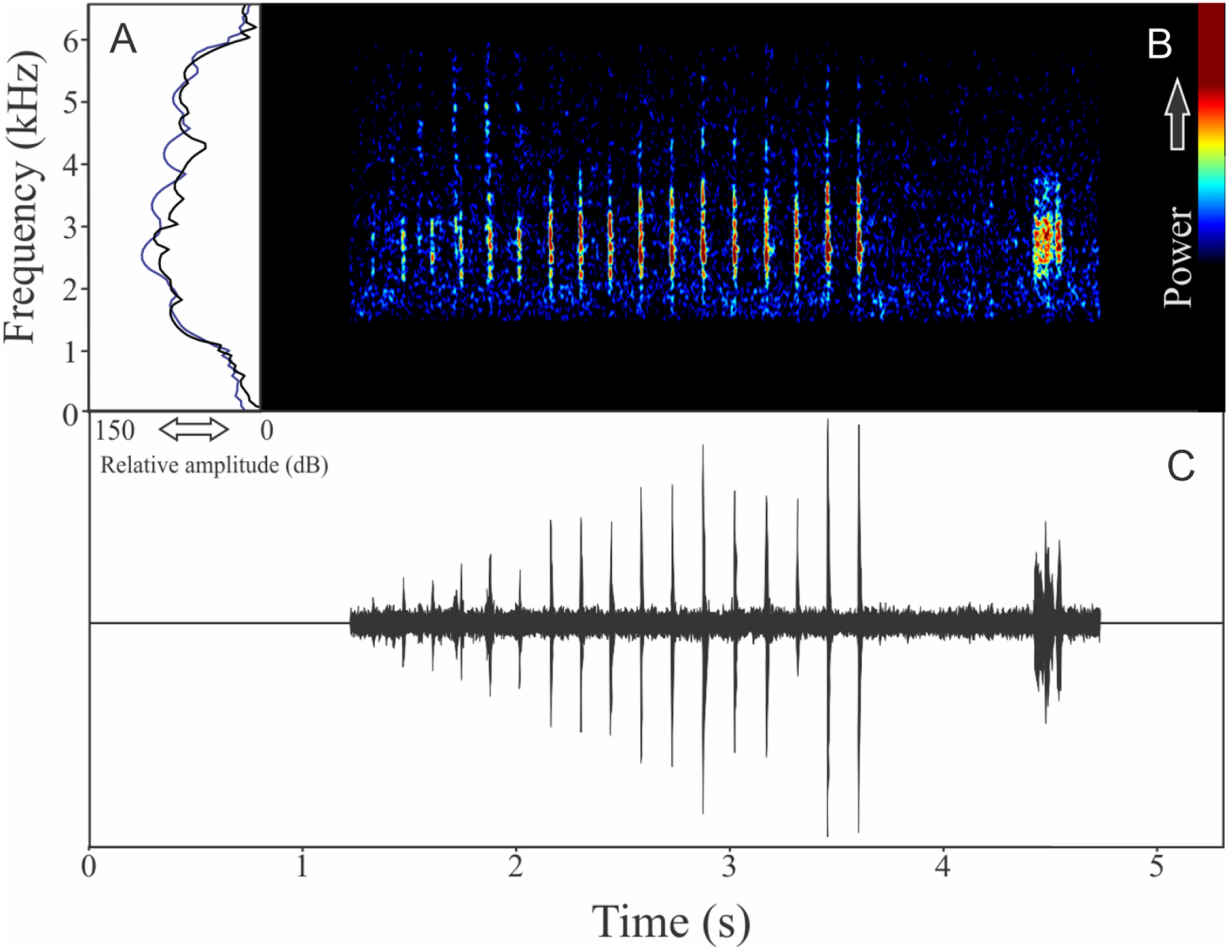
Amplitude spectra, spectrogram, and waveform of the advertisement call of *Ololygon flavoguttatus*. (A) Amplitude spectra (taken near to the midst of the call—note A is the blue line and note B is the black line), (B) spectrogram, and (C) waveform of the advertisement call of *Ololygon flavoguttatus* from Cataguases, state of Minas Gerais, Southeastern Brazil. Spectrogram window with DFT of 4096, grid spacing of 10.8 Hz and overlap of 75%.

We found two different vocalizations: (1) a sequence of harmonic notes (click-like) with a discrete interval and (2) a non-harmonic note composed by fused pulses (Fig. 15). We suggest that the first vocalization is the advertisement call, while the note composed by fused pulses is aggressive. These vocalizations may be emitted isolated or in combination (mixed calls). When combined, the advertisement call always anticipates the aggressive call. In these occasions, the interval between call types is 0.77 ± 0.34 s (ranging from 0.48 to 1.67 s, *n* = 29). The recorded male has a call rate of nine calls/min. The advertisement call has a variation of one to 25 pulses and duration of 1.70 ± 1.07 s (ranging from 0.03 to 3.66 s, *n* = 19), with rise time to the maximum amplitude of 1.42 ± 0.99 s (ranging from 0.01 to 3.13 s, *n* = 19). The range frequency in advertisement calls is 1,763 ± 719 Hz, with minimum frequency averaging 2,149 ± 222 Hz (ranging from 1,766 to 2,541 Hz, *n* = 19), maximum frequency of 3,912 ± 523 Hz (ranging from 3,273 to 5,039 Hz, *n* = 19), and peak dominant frequency (= fundamental frequency) of 2,643 ± 146 Hz (ranging from 2,283 to 2,972 Hz, *n* = 19). The second harmonic is up to four kHz. The aggressive call has a variation of one to seven pulses and duration of 0.19 ± 0.07 s (ranging from 0.08 to 0.41 s, *n* = 22), with rise time to the maximum amplitude of 0.14 ± 0.08 s (ranging from 0.05 to 0.38 s, *n* = 22). The range frequency of aggressive calls is 1,159 ± 250 Hz, with minimum frequency averaging 2,300 ± 144 Hz (ranging from 2,110 to 2,584 Hz, *n* = 22), maximum frequency of 3,459 ± 128 Hz (ranging from 3,230 to 3,704 Hz, *n* = 22), and peak dominant frequency of 2,786 ± 192 Hz (ranging from 2,498 to 3,230 Hz, *n* = 22).

*Ololygon tripui* (Lourenço, Nascimento & Pires, 2010)

**Figure 16:**
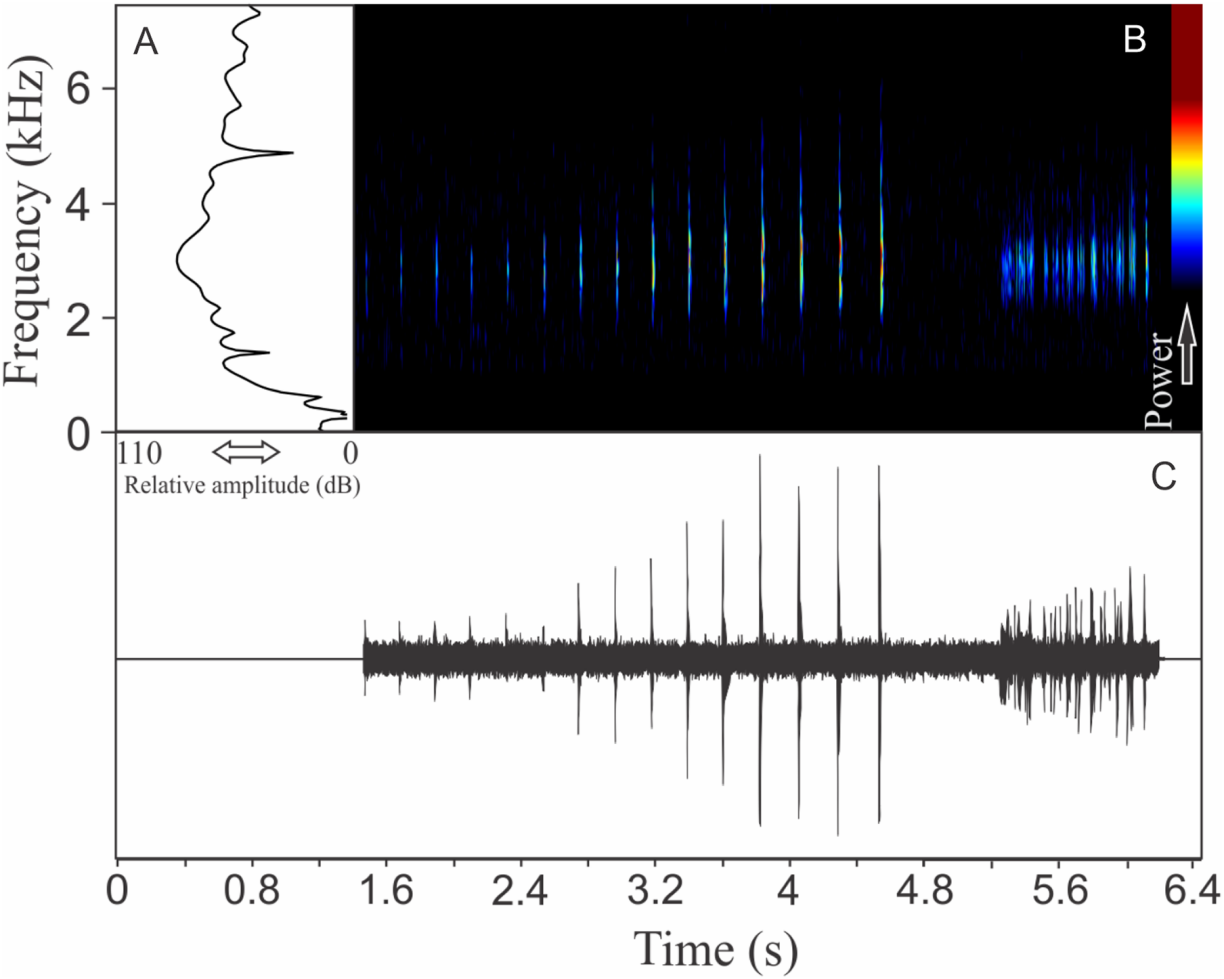
Amplitude spectra, spectrogram, and waveform of the advertisement call of *Ololygon tripui*. (A) Amplitude spectra (taken near to the midst of the first call), (B) spectrogram, and (C) waveform of the advertisement call of *Ololygon tripui* from Alto-Caparaó, state of Minas Gerais, Southeastern Brazil. Spectrogram window with DFT of 4096, grid spacing of 10.8 Hz and overlap of 75%.

Similar to what we found for *O. flavoguttata*, this species has two different vocalizations: (1) a sequence of non-pulsed short notes (eight to 12 notes, *n* = 17, males = 2) and (2) a long non-harmonic note with fused pulses (30–44 pulses, *n* = 2, males = 2) (Fig. 16). We suggest that the first vocalization is the advertisement call, while the note composed by fused pulses is an aggressive call. These vocalizations may be emitted isolated or in combination (mixed calls). When combined, the advertisement call always precedes the aggressive call. The advertisement call has duration of 3.22 ± 0.15 s (ranging from 2.2 to 4.1 s, *n* = 5, males = 2). Each note in the advertisement call has duration of 0.021 ± 0.001 s (ranging from 0.017 to 0.029 s, *n* = 17, males = 2), with rise time to the maximum amplitude of 0.0025 ± 0.0022 s (ranging from 0.000 to 0.006 s, *n* = 17, males = 2). The range frequency in advertisement calls is 1,588 ± 78 Hz, with minimum frequency averaging 2,360 ± 89 Hz (ranging from 2,282 to 2,540 Hz, *n* = 17, males = 2), maximum frequency of 3,948 ± 11 Hz (ranging from 3,703 to 4,220 Hz, *n* = 17, males = 2), and peak dominant frequency of 3,035 ± 12 Hz (ranging from 2,813 to 3,273 Hz, *n* = 17, males = 2). The aggressive call has duration of 0.502 ± 0.531 s (ranging from 0.126 to 0.877 s, *n* = 2, males = 2), with rise time to the maximum amplitude of 0.413 ± 0.509 s (ranging from 0.053 to 0.773 s, *n* = 2, males = 2). The range frequency of aggressive calls is 5,958 ± 752 Hz, with minimum frequency averaging 2,002 ± 274 Hz (ranging from 1,809 to 2,196 Hz, *n* = 2, males = 2), maximum frequency of 7,961 ± 478 Hz (ranging from 7,623 to 8,300 Hz, *n* = 2, males = 2), and peak dominant frequency of 3,100 ± 121 Hz (ranging from 3,014 to 3,186 Hz, *n* = 2, males = 2).

PHYLLOMEDUSIDAE
*Phasmahyla cochranae* (Bokermann, 1966)

**Figure 17:**
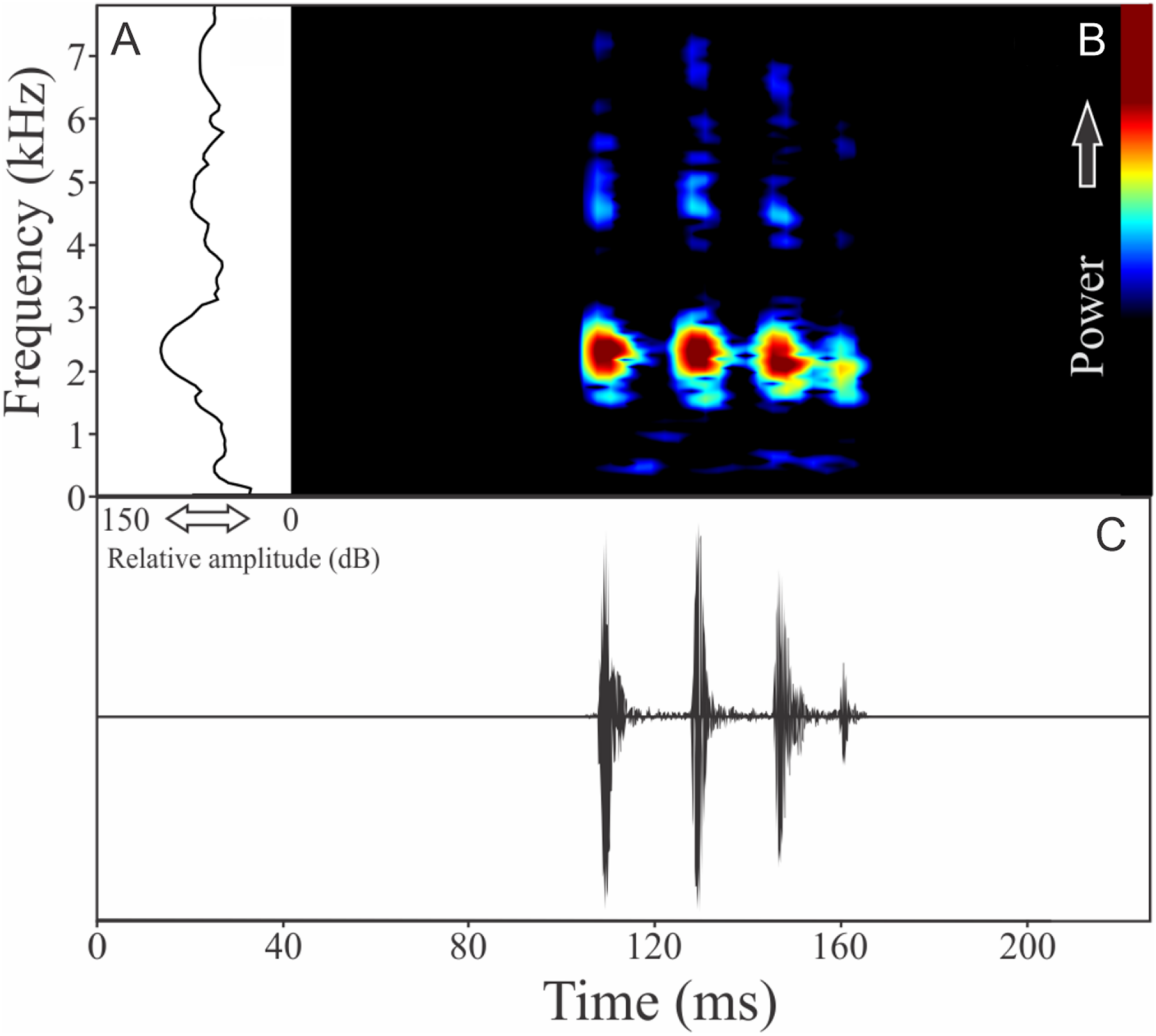
Amplitude spectra, spectrogram, and waveform of the advertisement call of *Phasmahyla cochranae*. (A) Amplitude spectra (taken near to the midst of the call), (B) spectrogram, and (C) waveform of the advertisement call of *Phasmahyla cochranae* from Jundiaí, state of São Paulo, Southeastern Brazil. Spectrogram window with DFT of 4096, grid spacing of 10.8 Hz and overlap of 75%.

The vocalization of *Phasmahyla cochranae* is a single harmonic note composed by three to four non-fused pulses (Fig. 17). Sometimes this note can be repeated as a series of four to eight units with regular intervals of 0.5 s. Call (note) duration is 0.056 ± 0.013 s (ranging from 0.040 to 0.079 s, *n* = 35, males = 2), with rise time to the maximum amplitude of 0.016 ± 0.014 s (ranging from 0.002 to 0.031 s, *n* = 35, males = 2). Males have a call rate varying between nine and 15 calls/min (*n* = 2). The range frequency is 614 ± 210 Hz, with minimum frequency averaging 1,621 ± 518 Hz (ranging from 1,206 to 2,024 Hz, *n* = 35, males = 2), maximum frequency of 2,236 ± 308 Hz (ranging from 1,895 to 2,498 Hz, *n* = 35, males = 2), and dominant frequency (= fundamental frequency) of 1,854 ± 569 Hz (ranging from 1,378 to 2,369 Hz, *n* = 35, males = 2). The second harmonic is about 4,700 Hz.

*Phasmahyla jandaia* (Bokermann & Sazima, 1978)

**Figure 18:**
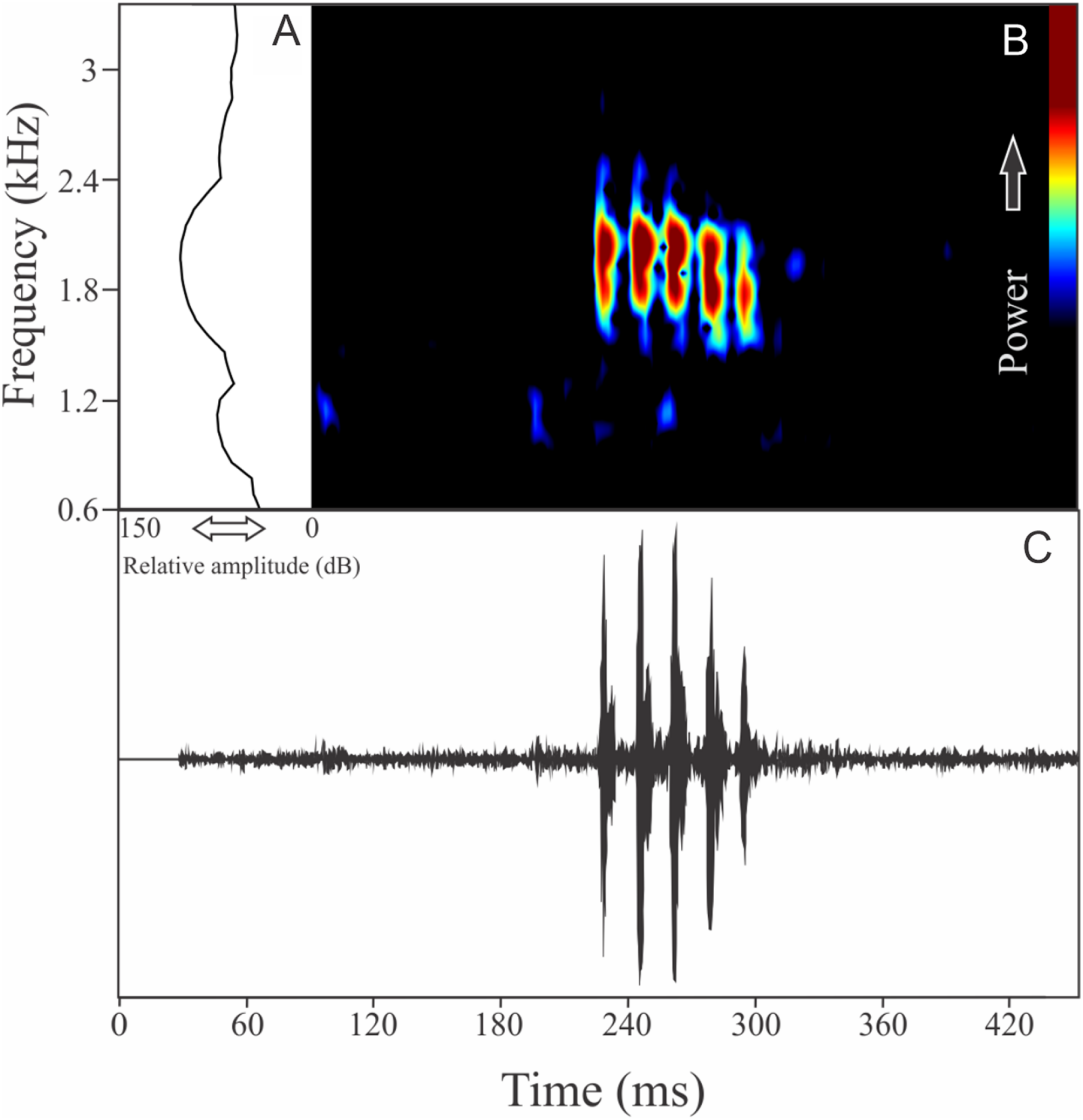
Amplitude spectra, spectrogram, and waveform of the advertisement call of *Phasmahyla jandaia*. (A) Amplitude spectra (taken near to the midst of the call), (B) spectrogram, and (C) waveform of the advertisement call of *Phasmahyla jandaia* from Congonhas, state of Minas Gerais, Southeastern Brazil. Spectrogram window with DFT of 4096, grid spacing of 10.8 Hz and overlap of 75%.

The vocalization of *Phasmahyla jandaia* is a single note composed by three to five non-fused pulses (Fig. 18). Call duration is 0.06 ± 0.02 s (ranging from 0.04 to 0.09 s, *n* = 7, males = 2), with rise time to the maximum amplitude of 0.021 ± 0.018 s (ranging from 0.002 to 0.040 s, *n* = 7, males = 2). Males have a call rate of three calls/min (*n* = 2, males = 2). The range frequency is 456 ± 5 Hz, with minimum frequency averaging 1,753 ± 3 Hz (ranging from 1,680 to 1,809 Hz, *n* = 7, males = 2), maximum frequency of 2,209 ± 3 Hz (ranging from 2,110 to 2,283 Hz, *n* = 7, males = 2), and dominant frequency of 2,019 ± 53 Hz (ranging from 1,895 to 2,110 Hz, *n* = 7, males = 2).

*Phrynomedusa appendiculata* (Lutz, 1925)

**Figure 19:**
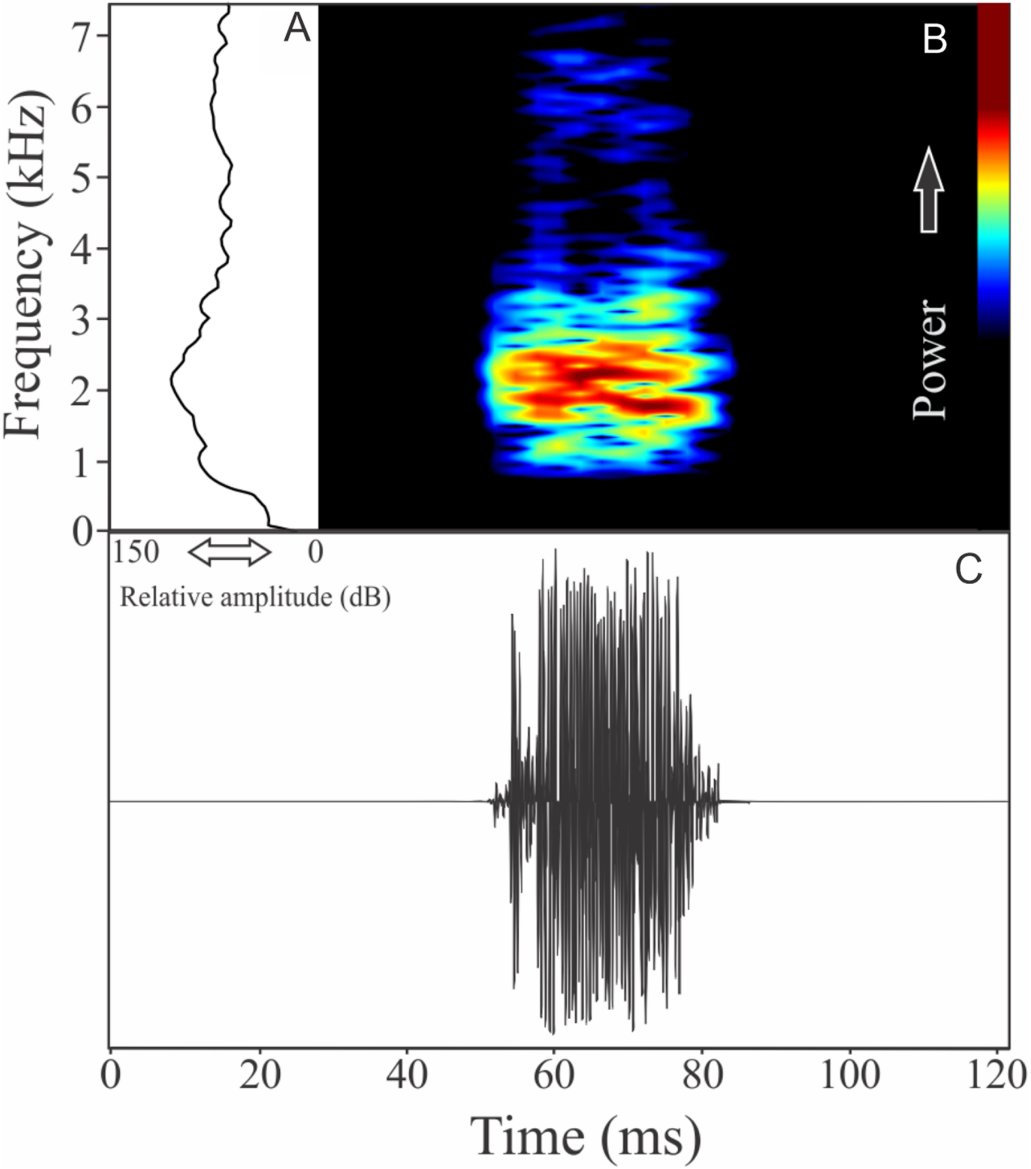
Amplitude spectra, spectrogram, and waveform of the advertisement call of *Phrynomedusa appendiculata*. (A) Amplitude spectra (taken near to the midst of the call), (B) spectrogram, and (C) waveform of the advertisement call of *Phrynomedusa appendiculata* from Paranapiacaba, Santo André, state of São Paulo, Southeastern Brazil. Spectrogram window with DFT of 4096, grid spacing of 10.8 Hz and overlap of 75%.

The vocalization of *Phrynomedusa appendiculata* is a single note composed by fused pulses (Fig. 19). Call duration is 0.03 ± 0.00 s (*n* = 2), with rise time to the maximum amplitude of 0.01 ± 0.01 s (ranging from 0.01 to 0.02 s, *n* = 2). The recorded male has a call rate of nine calls/min (*n* = 1). The range frequency is 991 ± 61 Hz, with minimum frequency ranging from 1,594 to 1,680 Hz (*n* = 2), maximum frequency of 2,627 Hz (*n* = 2), and peak dominant frequency ranging from 1,766 to 2,196 Hz (*n* = 2).

*Phyllomedusa iheringii* Boulenger, 1885

**Figure 20:**
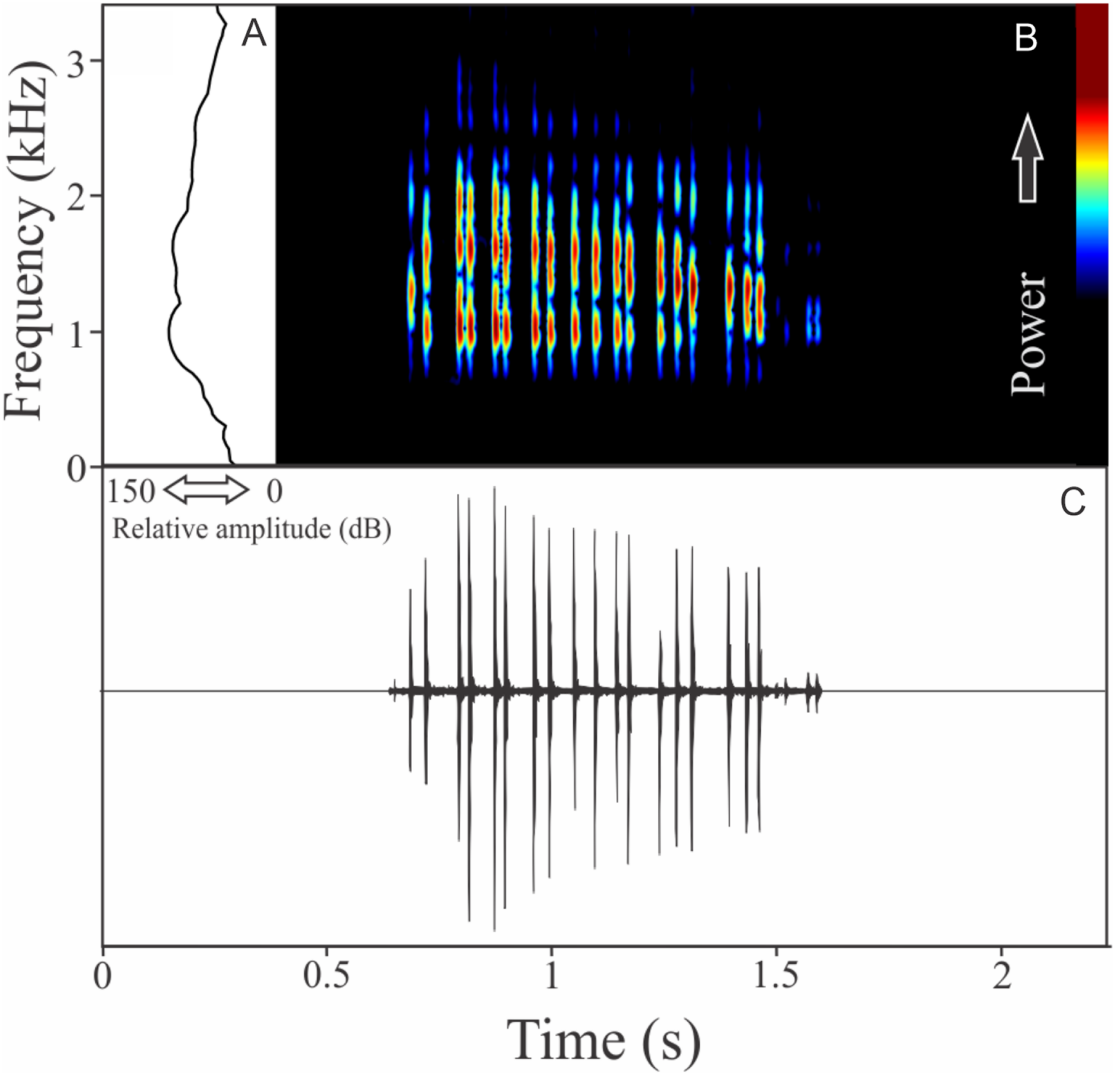
Amplitude spectra, spectrogram, and waveform of the advertisement call of *Phyllomedusa iheringii*. (A) Amplitude spectra (taken near to the midst of the call), (B) spectrogram, and (C) waveform of the advertisement call of *Phyllomedusa iheringii* from Bagé, state of Rio Grande do Sul, Southern Brazil. Spectrogram window with DFT of 4096, grid spacing of 10.8 Hz and overlap of 75%.

The vocalization of *Phyllomedusa iheringii* is a single note composed of seven to 36 non-fused pulses (Fig. 20). Call duration is 0.80 ± 0.59 s (ranging from 0.27 to 1.87 s, *n* = 18), with rise time to the maximum amplitude of 0.27 ± 0.26 s (ranging from 0.08 to 1.06 s, *n* = 18). The recorded male has a call rate of six calls/min (*n* = 1). The range frequency is 1,074 ± 219 Hz, with minimum frequency averaging 975 ± 41 Hz (ranging from 818 to 991 Hz, *n* = 18), maximum frequency of 2,031 ± 196 Hz (ranging from 1,766 to 2,670 Hz, *n* = 18), and dominant frequency of 1,290 ± 202 Hz (ranging from 1,034 to 1,938 Hz, *n* = 18).

*Pithecopus rusticus* (Bruschi et al., 2015)

**Figure 21:**
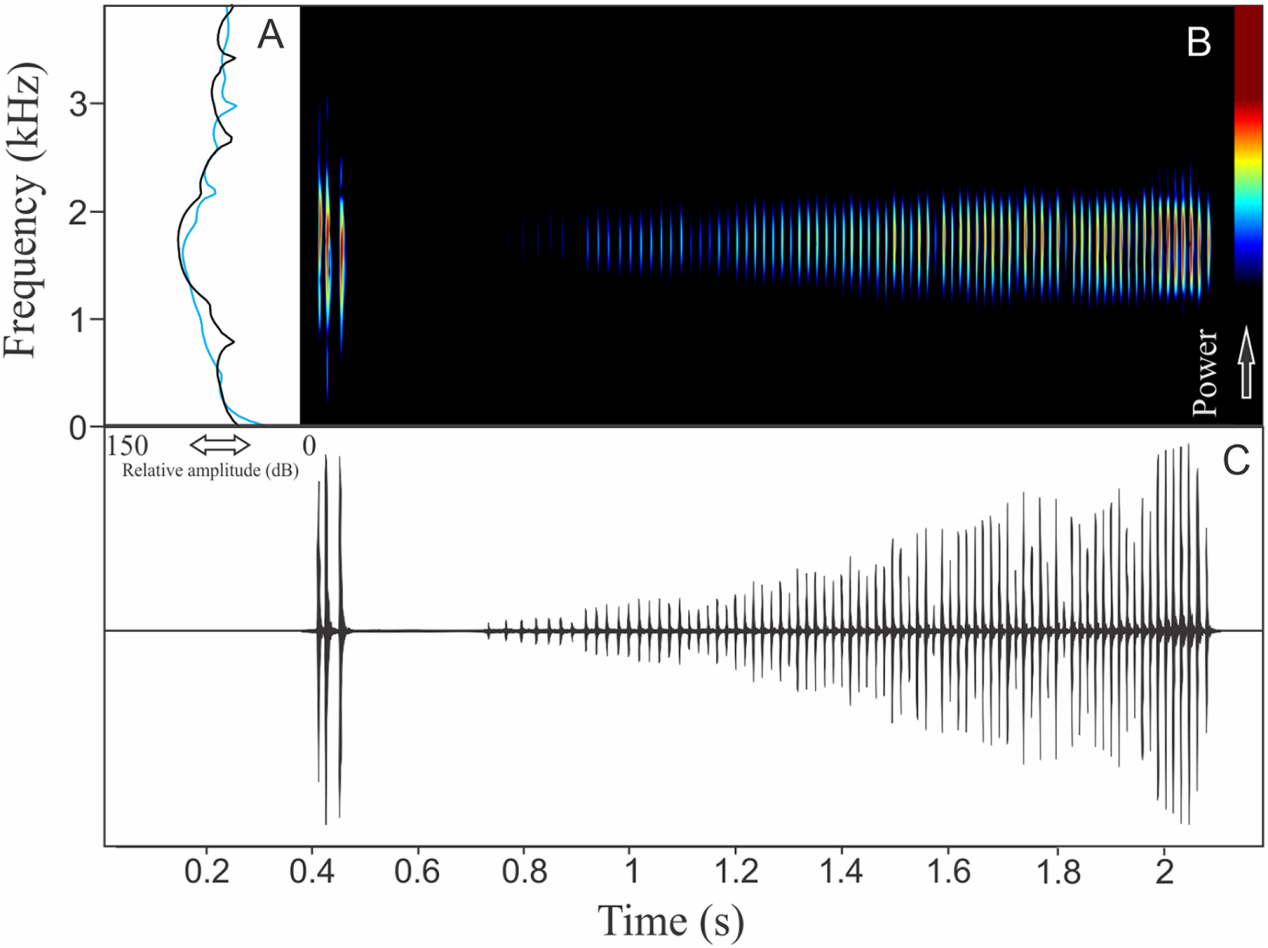
Amplitude spectra, spectrogram, and waveform of the advertisement call of *Pithecopus rusticus*. (A) Amplitude spectra (taken near to the midst of the call—short call is the blue line and long call is the black line), (B) spectrogram, and (C) waveform of the advertisement call of *Pithecopus rusticus* from Água Doce, state of Santa Catarina, Southern Brazil. Spectrogram window with DFT of 4096, grid spacing of 10.8 Hz and overlap of 75%.

The vocalization of *Pithecopus rusticus* has two acoustic units: (1) a short pulsed note with duration of 0.045 ± 0.002 s and (2) a long pulsed note with duration of 1.196 ± 0.308 s (Fig. 21). These notes may be combined or emitted in an isolated way. When combined the interval between notes is 4 ± 0.87 s (*n* = 17, males = 3). The mean call rate was 14 ± 2 calls/min (males = 3). The short note has two to three pulses with rise time to the maximum amplitude of 0.011 ± 0.008 s (ranging from 0.003 to 0.036 s, *n* = 17, males = 3) and range frequency of 900 Hz, with minimum frequency of 1,112 ± 81 Hz (ranging from 302 to 1,249 Hz, *n* = 17, males = 3), mean maximum frequency of 2,012 ± 8 Hz (ranging from 1,981 to 2,110 Hz, *n* = 17, males = 3), and a peak dominant frequency of 1,623 ± 64 Hz (ranging from 1,249 to 1,852 Hz, *n* = 17, males = 3). The long note has 50–80 pulses with rise time to the maximum amplitude of 1.126 ± 0.300 s (ranging from 0.784 to 1.345 s, *n* = 3) and range frequency of 631 Hz, with minimum frequency of 1,363 ± 108 Hz (ranging from 1,249 to 1,464 Hz, *n* = 3), mean maximum frequency of 1,995 ± 138 Hz (ranging from 1,895 to 2,153 Hz, *n* = 3), and peak of dominant frequency of 1,751 ± 49 Hz (ranging from 1,723 to 1,809 Hz, *n* = 3).

## Discussion

About half of the Brazilian amphibians occur in the Atlantic Forest (Toledo & Batista, 2012; Haddad et al., 2013; Segalla et al., 2014). Even though the rate of call descriptions has increased exponentially in the last years, a recent review (Guerra et al., 2018) showed that most Brazilian species with undescribed advertisement calls are concentrated in the Amazon Basin and mainly in the Atlantic Forest. Many species have restricted distribution or are rare (Toledo et al., 2014) and these factors together potentially affect the high number of species that remain with calls to be described.

Below we present a general view about the acoustics knowledge inside each genus or species group with call described in this paper:

### *Ischnocnema lactea* species group

*Ischnocnema concolor* and *I. melanopygia* are members of the *I. lactea* species series (Padial, Grant & Frost, 2014; Taucce et al., 2018), and both species have calls with harmonic notes, differing strictly in note duration and spectral band. While some species as *I. concolor* and *I. vizitoi* have calls composed only by one non-pulsed harmonic note, *I. melanopygia* may have calls formed by a sequence of three to five of these notes (Martins & Haddad, 2010). Calls of *I. lactea* are composed by one multipulsed note (Silva-Soares et al., 2018) and other species of the species series, as *I. nigriventris* and *I. randorum* have calls composed by more than one note with no apparent harmonics (Berneck, Targino & Garcia, 2013; Heyer et al., 1990). Rocha et al. (2017) and Silva-Soares et al. (2018) provided a detailed comparison of acoustic properties among species.

### Genus *Dendrophryniscus*

The genus *Dendrophryniscus* is composed of 16 species, occurring in the Atlantic Forest (Frost, 2019). All of them with undescribed calls. Like other congeneric species, *D. berthalutzae* is a bromeliad phytotelmata specialist (Malagoli et al., 2017). Further effort should be employed to obtain recordings of different species trying to improve the taxonomic resolution for this genus. As it is difficult to record this species, maybe the use of autonomous recorders can contribute for obtaining such data. The sequence of pulsed notes that compose the vocalization in *D. berthalutzae* can be a conserved feature of the Bufonidae family (Alonso & Rodríguez, 2003; Martin, 1972).

### Genus *Melanophryniscus*

The vocalizations of *M. alipioi*, *M. moreirae*, and *M. vilavelhensis* are equally complex, with two different types of notes. As observed for *M. moreirae*, many species of the genus combine isolate notes with a sequence of notes (Duré, Schaefer & Kehr, 2015). The social function of these call sections is unknown, but it is possible that they are advertisement and aggressive signals. Playback experiments should be performed to elucidate such functions. However, *M. alipioi* and *M. vilavelhensis* have two different note sequences (segments) mainly determined by variations in silence interval, being that the introductory segment has notes with longer duration and a larger interval among them than the notes in the main segment. This configuration is similar to *M. atroluteus*, *M. pachyrhynus*, *M. krauczuki*, *M. montevidensis*, and *M. dorsalis* (Caldart, dos Santos & Maneyro, 2013).

### Genus *Ceratophrys*

Among the Neotropical horned frogs, *Ceratophrys* is the most diverse genus of the family Ceratophryidae (Frost, 2019); however, they are the less known regarding acoustic descriptions (Lescano, 2011; Zaidan & Leite, 2012). Vocalizations composed by a single note with multiple pulses seem to be a common feature for the Ceratophryidae family (Lescano, 2011; Zaidan & Leite, 2012). The vocalization of *Ceratophrys aurita* has the lowest frequency among the species of the genus with calls already described (see a complete comparison of acoustic traits in Zaidan & Leite (2012)).

### Genus *Cycloramphus*

Vocalizations in the genus *Cycloramphus* may be emitted in three configurations regarding notes and pulses organization: (1) one non-pulsed note, (2) one pulsed note, and (3) a sequence of unpulsed or pulsed notes (Lingnau et al., 2008; Lima et al., 2010). *Cycloramphus granulosus* and *Cycloramphus izecksohni* emits a type (2) call, however, the second species can combine two pulsed notes as a call unit. Many species of this genus reproduce in small waterfalls in the Atlantic Forest (Heyer, 1983) and have to deal with an intense low frequency background noise. It is possible that spectral call traits have been modulated by such environmental condition and this should be a subject of interest in future research involving this taxonomic group. Lima et al. (2010) presented a detailed comparison of calls traits among different species.

### Genus *Zachaenus*

*Zachaenus* is a genus of frogs endemic to the Atlantic Forest, composed by only two species: *Z. carvalhoi* Izecksohn and *Z. parvulus* (Frost, 2019). Both species emit vocalizations composed by one or more multipulsed notes (one to six in *Z. carvalhoi*) and a call duration of 0.12 to 0.40 s (Guimarães, Lacerda & Feio, 2013; Mollo-Neto et al., 2016; Guedes et al., 2019). However, the vocalization of *Z. carvalhoi* shows higher dominant frequency (above 2,000 Hz) (Guimarães, Lacerda & Feio, 2013; Mollo-Neto et al., 2016). Pulses per note vary between two and 12 in *Z. carvalhoi* (Guimarães, Lacerda & Feio, 2013; Mollo-Neto et al., 2016).

### *Boana pulchella* species group

Vocalizations of species from *Boana pulchella* group are very diverse, varying from simple calls with isolated notes, to calls composed of several identical notes, and complex calls (mixing different notes) (Heyer et al., 1990; Köhler et al., 2010; Guerra, Lingnau & Bastos, 2017). Simple calls with isolated notes, as in *Boana guentheri*, are found in *Boana balzani*, *Boana botumirim*, *Boana curupi*, *Boana ericae*, *Boana callipleura*, and *Boana polytaenia* (Garcia, Faivovich & Haddad, 2007; Garcia & Haddad, 2008; Caramaschi, Cruz & Nascimento, 2009; Köhler et al., 2010; Pinheiro, Pezzuti & Garcia, 2012). *Boana marianitae* has calls composed by a sequence of repeated similar notes (Köhler et al., 2010), while other species as *Boana bandeirantes*, *Boana caingua*, *Boana jaguariaivensis*, *Boana leptolineata*, *Boana latistriata*, and *Boana riojana* have complex calls (Heyer et al., 1990; Köhler et al., 2010; Guerra, Lingnau & Bastos, 2017; De Luna-Dias & De Carvalho-e-Silva, 2019). The vocalization of *Boana leptolineata* is structurally very similar to *Boana bandeirantes* and *Boana jaguariaivensis* considering the divergence of notes, however, notes “A” in *Boana leptolineata* and *Boana bandeirantes* have more fused pulses than the note “A” in *Boana jaguariaivensis* (Guerra, Lingnau & Bastos, 2017). Batista et al. (2015) presents a table comparing acoustic traits among several species of *Boana pulchella* group.

### Genus *Bokermannohyla*

The genus *Bokermannohyla* has 32 species, 19 of them belonging to the *Bokermannohyla circumdata* species group (Frost, 2019). Despite recent effort describing several new species in the last 10 years, this group needs more attention to improve its taxonomic resolution by an integrative view (Faivovich et al., 2005). Among the Atlantic Forest species of the *Bokermannohyla circumdata* species group, only *Bokermannohyla caramaschii* remains with undescribed calls, since *Bokermannohyla izecksohni* is considered voiceless (Toledo et al., 2014). Vocalizations in this group of species may vary between isolated pulsed notes to complex calls with different notes (Gaiga et al., 2013). Similar to the vocalization of *Bokermannohyla gouveai* are the calls of *Bokermannohyla circumdata*, which emit a single and harmonic note (De Carvalho, Giaretta & Magrini, 2012). Many other species of the group, as *Bokermannohyla astartea*, *Bokermannohyla luctuosa*, and *Bokermannohyla nanuzae* have two types of notes in their calls (Heyer et al., 1990; Napoli & Caramaschi, 2004; De Carvalho, Giaretta & Magrini, 2012), possibly with different social functions. Gaiga et al. (2013) provide a thorough comparison of acoustic traits among species.

### *Ololygon catharinae* species group

Many species of the *O. catharinae* group may show mixed calls (possible advertisement + aggressive calls) (Hepp, Lourenço & Pombal, 2017), as we describe for *O. flavoguttata* and *O. tripui*. Species may have calls composed by click-like, long and short squawk-like notes as classified by Hepp, Lourenço & Pombal (2017). Squawk-like notes, supposedly phylogenetically conserved among *Ololygon* species (Bang & Giaretta, 2017), were not identified in the vocalization of *O. flavoguttata* and *O. tripui*. Considering the *O. catharinae* species group restricted to the Atlantic Forest, now the calls of 21 species are formally described (Hepp, Lourenço & Pombal, 2017; present study), with the vocalizations of the following species remaining to be described: *O. ariadne*, *O. brieni*, *O. carnevallii*, *O. jureia*, *O. kautskyi*, *O. melanodactyla*, *O. muriciensis*, *O. obtriangulata*, and *O. skuki*. Hepp, Lourenço & Pombal (2017) presented a detailed comparison of acoustic traits among species of the *O. catharinae* group.

### Genus *Phasmahyla*

Before our work, vocalizations of species in the genus *Phasmahyla* was only known for three species (out of eight): *Phasmahyla spectabilis* (Dias et al., 2011), *Phasmahyla timbo* (Cruz, Napoli & Fonseca, 2008), and *Phasmahyla lisbella* (Pereira et al., 2018). Calls of these three species are very similar to those of *Phasmahyla cochranae* and *Phasmahyla jandaia*. All five species have calls composed by a single note with non-fused pulses (Cruz, Napoli & Fonseca, 2008; Dias et al., 2011; Pereira et al., 2018). *Phasmahyla jandaia* has the longest notes (achieving 0.09 s), while *Phasmahyla spectabilis* and *Phasmahyla timbo* have notes around 0.03 s (Cruz, Napoli & Fonseca, 2008; Dias et al., 2011). *Phasmahyla lisbella* shows high variation in note duration, from 0.007 to 0.087 s (Pereira et al., 2018). Probably this difference is due to the larger number of pulses in *Phasmahyla jandaia* than in other species. Harmonic structure is only visible in *Phasmahyla cochranae*. Dominant frequency in all species ranges between 1,700 and 2,200 Hz. *Phasmahyla timbo* has a call with lower frequencies compared to the other species. New recording efforts should be made to obtain recordings of the other species without calls described, as *Phasmahyla exilis* and *Phasmahyla guttata*.

### Genus *Phrynomedusa*

The current knowledge of acoustics in this genus, before our work, was limited to two species (out of six): *Phrynomedusa marginata* (Weygoldt, 1991) and *Phrynomedusa dryade* (Baêta et al., 2016). The last species was recently described by Baêta et al. (2016), but Weygoldt (1991) presented only superficial acoustic data for a male *Phrynomedusa marginata* recorded from a terrarium. Summed with *Phrynomedusa appendiculata* both species have calls composed by a single note with short duration (Weygoldt, 1991), while *Phrynomedusa dryade* has longer calls composed by a series of pulsed notes (Baêta et al., 2016). Call descriptions still remain to be known for *Phrynomedusa bokermanni*, *Phrynomedusa fimbriata*, and *Phrynomedusa vanzolini*, although, *Phrynomedusa fimbriata* is considered as an extinct taxon (IUCN, 2017).

### *Phyllomedusa burmeisteri* species group

The *Phyllomedusa burmeisteri* group is represented by five species (Faivovich et al., 2010) and the vocalizations, now (including *Phyllomedusa iheringii*), are totally described. Calls composed by a single note with non-fused pulses are common to all species (Haddad, Pombal & Batistic, 1994; Abrunhosa & Wogel, 2004; Silva-Filho & Juncá, 2006). *Phyllomedusa iheringii* has longer calls (0.80 s) than other species, which vary among 0.20 and 0.40 s (Haddad, Pombal & Batistic, 1994; Abrunhosa & Wogel, 2004; Silva-Filho & Juncá, 2006). According to Haddad, Pombal & Batistic (1994), advertisement calls of *Phyllomedusa distincta* and *Phyllomedusa tetraploidea* are spectrally indistinguishable, they occupy a range frequency of 700–2,500 Hz, making the bioacoustics an apparently weak feature for species recognition (Köhler et al., 2017). Possibly, this high similarity in sexual signals among different species promote extensive cases of hybridization between these sympatric species (Haddad, Pombal & Batistic, 1994). De Andrade et al. (2018) compared calls of *Phyllomedusa burmeisteri* and *Phyllomedusa bahiana* and also defined that calls of these species cannot be distinguished by qualitative or quantitative acoustic properties. However, a focused and standardized study based in a robust data set for comparing the vocalizations among species of the *Phyllomedusa burmeisteri* group is still necessary for a more reliable understanding.

### Genus *Pithecopus*

The genus *Pithecopus* comprises 11 species, with only three occurring in the Atlantic forest: *Pithecopus nordestinus*, *Pithecopus rohdei*, and *Pithecopus rusticus* (Frost, 2019). All species belong to the *Pithecopus hypochondrialis* group (Faivovich et al., 2005). Males of many species in this taxonomic group have calls composed by two different notes as described in *Pithecopus rusticus*, then an acoustic repertoire with short and long pulsed notes are exhibited by *Pithecopus ayeaye*, *Pithecopus azureus*, *Pithecopus centralis*, *Pithecopus hypochondrialis*, *Pithecopus nordestinus*, and *Pithecopus rohdei* (Guimarães et al., 2001; Wogel, Abrunhosa & Pombal, 2004; Brandão et al., 2009; Vilaça, Silva & Solé, 2011; Nali, Borges & Prado, 2015; Haga et al., 2017b). It is possible that these notes have different social function, with the short note being an advertisement signal and long notes emitted in an aggressive context. However, we suppose that such assumptions still should be tested using playback experiments. Advertisement calls of *Pithecopus araguaius* have only one acoustic unit as an isolated pulsed note, similar to the longer note by *Pithecopus rusticus*, but with less pulses (five to eight pulses) (Haga et al., 2017a). In *Pithecopus palliatus* the advertisement call is one or two notes with indistinct pulses (Köhler & Lötters, 1999). Calls of *Pithecopus megacephalus* and *Pithecopus oreades* remain undescribed.

## Conclusion

Our work extends the acoustic knowledge for anuran species from the Atlantic Forest, describing the vocalization of 20 species. Despite such progress, a further effort increasing the sample of recorded males for species represented by only one male in our analysis should improve the perception of call variation in these species.

Descriptions of hylid calls have been the focus of many recent papers, probably because it is the most diverse family of frogs in the Atlantic Forest. However, the families Phyllomedusidae and Cycloramphidae were well represented in our results despite the fact that these families are not as diverse as Hylidae. Despite there being 163 species of Atlantic anurans with calls not described yet, our work represents an important step in providing data for an integrative taxonomy and the best knowledge of such rich biodiversity. A future geographical analysis linking the distributions of these species should be helpful, which may point us in new directions to reduce this gap. Finally, we argue that sound files should always be deposited in sound archives, in order to promote the rapid access to such biodiversity component, neglected even by nowadays taxonomists (Toledo, Tipp & Márquez, 2015).

## Supplemental Information

10.7717/peerj.7612/supp-1 Supplemental Information 1 List of Atlantic forest anuran species (described up to April 2019) lacking the description of its advertisement calls. **Table S1**. List of Atlantic forest anuran species (described up to April 2019) lacking the description of its advertisement calls.

## Additional Information and Declarations

### Data Availability

The following information was supplied regarding data availability: Raw data is available at Lucas Rodriguez Forti. (2019). Notes on vocalizations of Brazilian amphibians iv (Data set). Zenodo. http://doi.org/10.5281/zenodo.3265941. All our audio files are available in the Fonoteca Neotropical Jacques Vielliard (FNJV, Unicamp, Campinas, https://www2.ib.unicamp.br/fnjv/): 36487–36496, 36497–36506, 36508, 12902, 12903, 37450–37453, 31783, 31785 and 32008, 33559, 31911, 31950, 34035, 31926, 33063, 30737–30739 and 31594, 36485, 36486, 32898 and 32389, 31990 and 31152, 36507 and 36509, 31849, 31156, 37454–37456. Specimens were not collected to this work.
