# Supplementary material for: Notes on vocalizations of Brazilian amphibians IV: advertisement calls of 20 Atlantic Forest frog species
Source: PeerJ. 2019 Sep 13;7:e7612. doi: 10.7717/peerj.7612 (PMC6746222; doi:10.7717/peerj.7612)
Supplement: Supplemental Information 1 — Table S1. List of Atlantic forest anuran species (described up to April 2019) lacking the description of its advertisement calls. [file peerj-07-7612-s001.docx]

**Table S1**. List of Atlantic forest anuran species (described up to April 2019) lacking the description of its advertisement calls.

| **Family / Species** |
| --- |
| **Brachycephalidae** |
| *Brachycephalus alipioi* Pombal & Gasparini 2006 |
| *Brachycephalus atelopoide* Miranda-Ribeiro 1920 |
| *Brachycephalus auroguttatus* Ribeiro, Firkowski, Bornschein & Pie 2015 |
| *Brachycephalus boticario* Pie, Bornschein, Firkowski, Belmonte-Lopes & Ribeiro 2015 |
| *Brachycephalus brunneus* Ribeiro, Alves, Haddad & Reis 2005 |
| *Brachycephalus bufonoides* Miranda-Ribeiro 1920 |
| *Brachycephalus coloratus* Ribeiro, Blackburn, Stanley, Pie & Bornschein 2017 |
| *Brachycephalus curupira* Ribeiro, Blackburn, Stanley, Pie & Bornschein 2017 |
| *Brachycephalus didactylus* (Izecksohn 1971) |
| *Brachycephalus ferruginus* Alves, Ribeiro, Haddad & Reis 2006 |
| *Brachycephalus fuscolineatus* Pie, Bornschein, Firkowski, Belmonte-Lopes and Ribeiro 2015 |
| *Brachycephalus garbeanus* Miranda-Ribeiro 1920 |
| *Brachycephalus guarani* Clemente-Carvalho, Giaretta, Condez, Haddad & Reis 2012 |
| *Brachycephalus izecksohni* Ribeiro, Alves, Haddad & Reis 2005 |
| *Brachycephalus leopardus* Ribeiro, Firkowski & Pie 2015 |
| *Brachycephalus margaritatus* Pombal & Izechsohn 2011 |
| *Brachycephalus mariaterezae* Bornschein, Morato, Firkowski, Ribeiro & Pie 2005 |
| *Brachycephalus nodoterga* Miranda-Ribeiro 1920 |
| *Brachycephalus pernix* Pombal, Wistuba & Bornschein 1998 |
| *Brachycephalus pombali* Alves, Ribeiro, Haddad & Reis 2006 |
| *Brachycephalus pulex* Napoli, Caramaschi, Cruz & Dias 2011 |
| *Brachycephalus toby* Haddad, Alves, Clemente-Carvalho & Reis 2010 |
| *Brachycephalus verrucosus* Ribeiro, Firkowski, Bornschein & Pie 2015 |
| *Brachycephalus vertebralis* Pombal 2001 |
| *Ischnocnema epipeda* (Heyer 1984) |
| *Ischnocnema erythromera* (Heyer 1984) |
| *Ischnocnema gehrti* (Miranda-Ribeiro 1926) |
| *Ischnocnema holti* (Cochran 1948) |
| *Ischnocnema karst* Canedo, Targino, Leire & Haddad 2012 |
| *Ischnocnema nanahallux* Brusquetti, Thome, Canedo, Condez & Haddad 2013 |
| *Ischnocnema octavioi* (Bokermann 1965) |
| *Ischnocnema paranaensis* (Langone & Segalla 1996) |
| *Ischnocnema pusilla* (Bokermann 1967) |
| *Ischnocnema spanios* (Heyer 1985) |
| *Ischnocnema surda* Canedo, Pimenta, Leite, and Caramaschi 2010 |
| *Ischnocnema venancioi* (Lutz 1958) |
| *Ischnocnema verrucosa* Reinhardt & Lütken 1862 |
| **Bufonidae** |
| *Dendrophryniscus carvalhoi* Izecksohn 1994 |
| *Dendrophryniscus krausae* Cruz & Fusinatto 2008 |
| *Dendrophryniscus leucomystax* Izecksohn 1968 |
| *Dendrophryniscus oreites* Recorder, Teixeira, Cassimiro, Camacho & Rodrigues 2010 |
| *Dendrophryniscus organensis* Carvalho-e-Silva, Mongin, Izechsohn & Carvalho-e-Silva, 2010 |
| *Dendrophryniscus proboscideus* (Boulenger 1882) |
| *Dendrophryniscus skuki* Caramaschi 2012 |
| *Dendrophryniscus stawiarskyi* Izechsohn 1994 |
| *Melanophryniscus admirabilis* Di-Bernardo, Maneyro & Grillo 2006 |
| *Melanophryniscus biancae* Bornschein, Baldo, Pie, Firkowski, Ribeiro & Corrêa 2015 |
| *Melanophryniscus cambaraensis* Braun & Braun 1979 |
| *Melanophryniscus macrogranulosus* Braun 1973 |
| *Melanophryniscus milanoi* Baldo, Bornschein, Pie, Firkowski, Ribeiro & Belmonte-Lopes 2015 |
| *Melanophryniscus peritus* Caramaschi & Cruz 2011 |
| *Melanophryniscus setiba* Peloso, Faivovich, Grant, Gasparini & Haddad 2012 |
| *Melanophryniscus simplex* Caramaschi & Cruz 2002 |
| *Melanophryniscus spectabilis* Caramaschi & Cruz 2002 |
| *Melanophryniscus xanthostomus* Baldo, Bornschein, Pie, Ribeiro, Firkowski & Morato 2015 |
| *Rhinella achavali* (Maneyro, Arrieta & de Sá 2004) |
| *Rhinella henseli* (Lutz 1934) |
| **Centrolenidae** |
| *Vitreorana baliomma* Pontes, Caramaschi & Pombal 2014 |
| *Vitreorana parvula* (Boulenger 1895) |
| **Craugastoridae** |
| *Haddadus plicifer* (Boulenger 1888) |
| *Holoaden bradei* Lutz 1958 |
| *Holoaden pholeter* Pombal, Siqueira, Dorigo, Vrcibradic & Rocha 2008 |
| *Holoaden suarezi* Martins & Zaher 2013 |
| *Pristimantis vinhai* (Bokermann 1975) |
| **Cycloramphidae** |
| *Cycloramphus acangatan* Verdade & Rodrigues 2003 |
| *Cycloramphus bandeirensis* Heyer 1983 |
| *Cycloramphus carvalhoi* Heyer 1983 |
| *Cycloramphus catarinensis* Heyer 1983 |
| *Cycloramphus diringshofeni* Bokermann 1957 |
| *Cycloramphus fuliginosus* Tschudi 1838 |
| *Cycloramphus lithomimeticus* Silva & Ouvernay 2012 |
| *Cycloramphus migueli* Heyer 1988 |
| *Cycloramphus mirandaribeiroi* Heyer 1983 |
| *Cycloramphus organensis* Weber, Verdade, Salles, Fouquet & Carvalo-e-Silva 2011 |
| *Cycloramphus stejnegeri* (Noble 1924) |
| *Thoropa saxatilis* Cocroft & Heyer 1988 |
| **Eleutherodactylidae** |
| *Adelophryne baturitensis* Hoogmoed, Borges & Cascon 1994 |
| *Adelophryne glandulata* Lourenço de Moraes, Ferreira, Fouquet & Bastos 2014 |
| *Adelophryne meridionalis* Santana, Fonseca, Neves & Carvalho 2012 |
| *Adelophryne michelin* Lourenço-de-Moraes, Dias, Mira-Mendes, Oliveira, Barth, Ruas, Vences, Solé, and Bastos, 2018 |
| *Adelophryne pachydactyla* Hoogmoed, Borges & Cascon 1994 |
| **Hemiphractidae** |
| *Fritziana tonimi* Walker, Gasparini & Haddad 2016 |
| *Fritziana ulei* (Miranda-Ribeiro 1926) |
| *Gastrotheca flamma* Juncá & Nunes 2008 |
| *Gastrotheca pulchra* Caramaschi & Rodrigues 2007 |
| **Hylidae** |
| *Aparasphenodon bokermanni* Pombal 1993 |
| *Aparasphenodon pomba* Assis, Santana, Silva, Quintela & Feio 2013 |
| *Aplastodiscus flumineus* (Cruz & Peixoto 1985) |
| *Boana cymbalum* (Bokermann 1963) |
| *Boana freicanecae* (Carnaval & Peixoto 2004) |
| *Boana guentheri* (Boulenger 1886) |
| *Boana secedens* (Lutz 1963) |
| *Bokermannohyla ahenea* (Napoli & Carmaschi 2004) |
| *Bokermannohyla caramaschii* (Napoli 2005) |
| *Bokermannohyla claresignata* (Lutz & Lutz 1939) |
| *Bokermannohyla langei* (Bokermann 1965) |
| *Dendropsophus dutrai* (Gomes & Peixoto 1996) |
| *Dendropsophus limai* (Bokermann 1962) |
| *Ololygon alcatraz* (Lutz 1973) |
| *Ololygon ariadne* (Bokermann 1967) |
| *Ololygon atrata* (Peixoto 1989) |
| *Ololygon brieni* (De Witte 1930) |
| *Ololygon carnevallii* Caramaschi & Kisteumacher 1989 |
| *Ololygon faivovichi* (Brasileiro, Oyamaguchi & Haddad 2007) |
| *Ololygon jureia* (Pombal & Gordo 1991) |
| *Ololygon kautskyi* Carvalho-e-Silva & Peixoto 1991 |
| *Ololygon longilinea* (Lutz 1968) |
| *Ololygon melanodactyla* (Lourenço, Luna & Pombal 2014) |
| *Ololygon melloi* Peixoto 1989 |
| *Ololygon muriciensis* (Cruz, Nunes & Lima 2011) |
| *Ololygon obtriangulata* (Lutz 1973) |
| *Ololygon skuki* (Lima, Cruz & Azevedo 2011) |
| *Ololygon trapicheiroi* (Lutz & Lutz 1954) |
| *Ololygon tupinamba* (Silva & Alves-Silva 2008) |
| *Phyllodytes brevirostris* Peixoto & Cruz 1988 |
| *Phyllodytes maculosus* Cruz, Feio & Cardoso 2007 |
| *Phyllodytes punctatus* Caramaschi, Silva & Britto-Pereira 1992 |
| *Sphaenorhynchus botocudo*Caramaschi, Almeida & Gasparini |
| *Sphaenorhynchus bromelicola* Bokermann 1966 |
| *Trachycephalus imitatrix* (Miranda-Ribeiro 1926) |
| *Trachycephalus lepidus* (Pombal, Haddad & Cruz 2003) |
| *Xenohyla eugenioi* Caramaschi 1998 |
| *Xenohyla truncata* (Izecksohn 1959) |
| **Hylodidae** |
| *Crossodactylus aeneus* Müller 1924 |
| *Crossodactylus boulengeri* (De Witte 1930) |
| *Crossodactylus dantei* Carcerelli & Caramaschi 1993 |
| *Crossodactylus dispar* Lutz 1925 |
| *Crossodactylus grandis* Lutz 1951 |
| *Crossodactylus lutzorum* Carcerelli & Caramaschi 1993 |
| *Hylodes mertensi* (Bokermann 1956) |
| **Leptodactylidae** |
| *Crossodactylodes itambe* Barata, Santos, Leite & Garcias 2013 |
| *Leptodactylus hylodes* (Reinhardt & Lütken 1862) |
| *Paratelmatobius lutzii* Lutz & Carvalho 1958 |
| *Paratelmatobius mantiqueira* Pombal & Haddad 1999 |
| *Physalaemus caete* Pombal & Madureira 1997 |
| *Physalaemus insperatus* Cruz, Cassini & Caramaschi 2008 |
| *Scythrophrys sawayae* (Cochran 1953) |
| **Microhylidae** |
| *Chiasmocleis alagoana* Cruz, Caramaschi & Freire 1999 |
| *Chiasmocleis altomontana* Forlani, Tonini, Cruz, Zaher & de Sá 2017 |
| *Chiasmocleis bicegoi* Miranda-Ribeiro, 1920 |
| *Chiasmocleis gnoma* Canedo, Dixo & Pombal 2004 |
| *Chiasmocleis migueli* Forlani, Tonini, Cruz, Zaher & de Sá 2017 |
| *Chiasmocleis quilombola* Forlani, Tonini & de Sá 2014 |
| *Chiasmocleis sapiranga* Cruz, Caramaschi & Napoli 2007 |
| *Chiasmocleis veracruz* Forlani, Tonini, Cruz, Zaher & de Sá 2017 |
| *Stereocyclops histrio* (Carvalho 1954) |
| *Stereocyclops palmipes* Caramaschi, Salles & Cruz 2012 |
| *Stereocyclops parkeri* (Wettstein 1934) |
| **Odontophrynidae** |
| *Proceratophrys belzebul* Dias, Amaro, Carvalo-e-Silva & Ridrigues 2013 |
| *Proceratophrys gladius* Mângia, Santana, Cruz &Feio 2014 |
| *Proceratophrys izecksohni* Dias, Amaro, Carvalo-e-Silva & Ridrigues 2013 |
| *Proceratophrys laticeps* Izecksohn & Peixoto 1981 |
| *Proceratophrys minuta* Napoli, Cruz, Abreu & Del Grande 2011 |
| *Proceratophrys phyllostomus* Izecksohn & Peixoto 1981 |
| *Proceratophrys schirchi* (Miranda-Ribeiro 1920) |
| *Proceratophrys subguttata* Izecksohnm Cruz & Peixoto 1999 |
| *Proceratophrys tupinamba* Prado & Pombal 2008 |
| **Phyllomedusidae** |
| *Phasmahyla cruzi* Carvalho-e-Silva, Silva & Carvalho-e-Silva 2009 |
| *Phasmahyla exilis* (Cruz 1980) |
| *Phasmahyla guttata* (Lutz 1924) |
| *Phrynomedusa bokermanni* Cruz 1991 |
| *Phrynomedusa fimbriata* Miranda-Ribeiro 1923 |
| *Phrynomedusa vanzolinii* Cruz 1991 |
|  |
